# Copper(II) NHC Catalyst for the Formation of Phenol from Arylboronic Acid

**DOI:** 10.3390/chemistry4020040

**Published:** 2022-06-07

**Authors:** Mitu Sharma, Bhupendra Adhikari, Raymond Femi Awoyemi, Amanda M. Perkins, Alison K. Duckworth, Bruno Donnadieu, David O. Wipf, Sean L. Stokes, Joseph P. Emerson

**Affiliations:** Department of Chemistry, Mississippi State University, Mississippi State, MS 39762-9573, USA;

**Keywords:** *N*-Heterocyclic ligands (NHC), pincer NHC, copper(II)-NHC complex, arylboronic acid, phenol, anisole

## Abstract

Arylboronic acids are commonly used in modern organic chemistry to form new C—C and C—heteroatom bonds. These activated organic synthons show reactivity with heteroatoms in a range of substrates under ambient oxidative conditions. This broad reactivity has limited their use in protic, renewable solvents like water, ethanol, and methanol. Here, we report our efforts to study and optimize the activation of arylboronic acids by a copper(II) *N*-heterocyclic carbene (NHC) complex in aqueous solution and in a range of alcohols to generate phenol and aryl ethers, respectively. The optimized reactivity showcases the ability to make targeted C—O bonds, but also identifies conditions where water and alcohol activation could be limiting for C—C and C—heteroatom bond-forming reactions. This copper(II) complex shows strong reactivity toward arylboronic acid activation in aqueous medium at ambient temperature. The relationship between product formation and temperature and catalyst loading are described. Additionally, the effects of buffer, pH, base, and co-solvent are explored with respect to phenol and ether generation reactions. Characterization of the new copper(II) NCN-pincer complex by X-ray crystallography, HR-MS, cyclic voltammetry, FT-IR and UV-Vis spectral studies is reported.

## 1. Introduction

**Figure 1.**
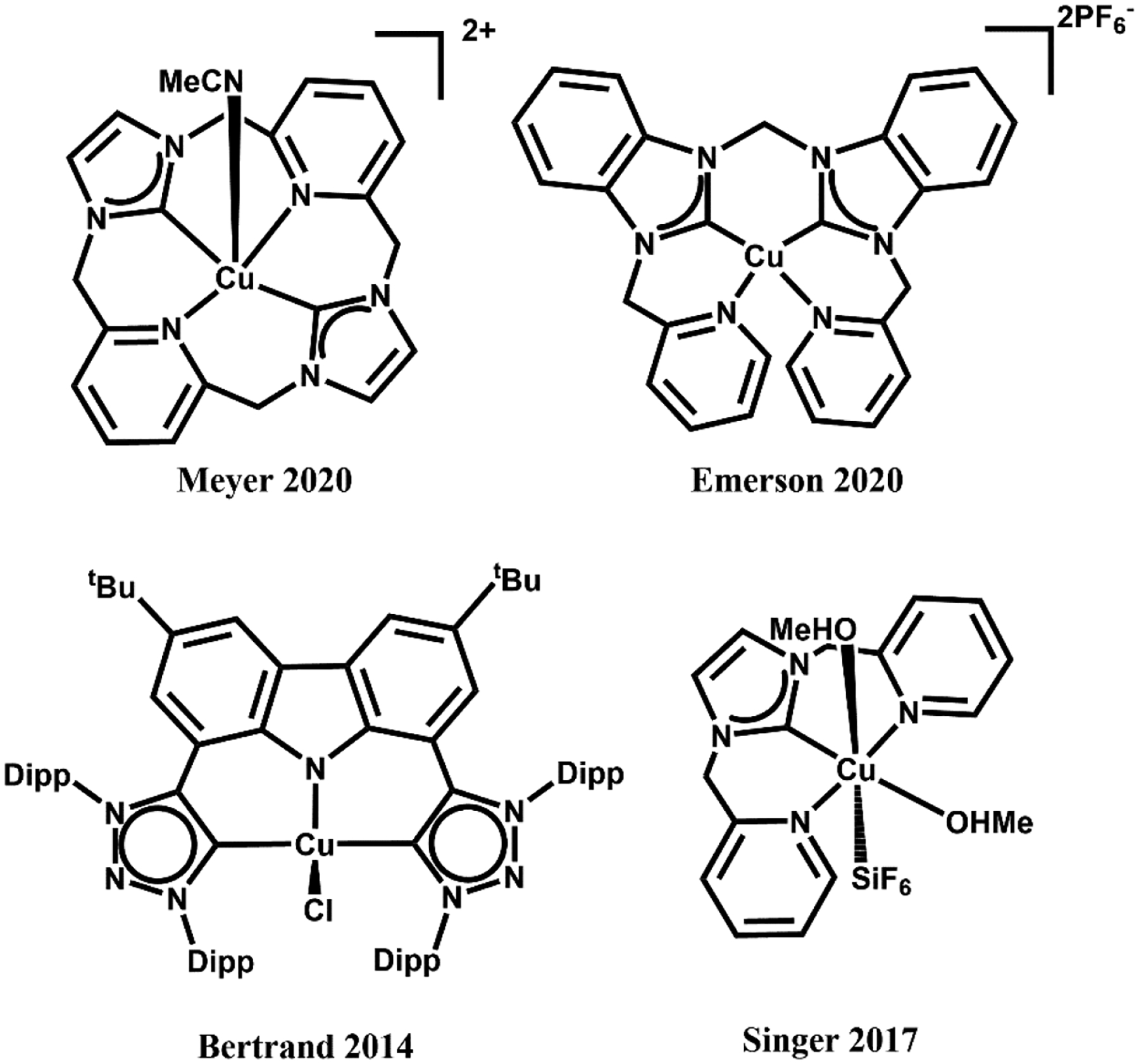
General structures of Cu^2+^ NHC complex.

Transition metal complexes of *N*-heterocyclic carbenes (NHCs) have acquired substantial attention because of their novel properties and extensive applications in catalysis [1–3]. In this respect, development of first-row transition metal catalysts for the formation of new C—C and C—heteroatom bonds has offered new vistas for development in the field of modern synthetic chemistry [4–6]. Copper-NHCs have attracted constant attention since the discovery of the first copper(I)-NHC complex in 1993 by Arduengo and co-workers [7]. Since then, a multitude of copper-NHC complexes have been synthesized to date [7–10]. However, the reports on copper(II)-NHCs are less abundant, with approximately 30 copper(II) NHC compounds having been reported [11]. The first copper(II)-NHC complex was reported by Meyer and co-workers in 2003, containing a tripodal NHC ligand [12]. Recently, there have been reports by Meyer and coworkers on copper(III)-NHC complex bearing pyridine groups forming a stable multidentate copper(III) coordination complex, [Cu(NCMe)][PF_6_]_2_[SbF_6_] [13,14]. Building on this system, our group reported the synthesis of a pyridyl-based tetradentate copper(II)-NHC complex, as shown in Figure 1, as coupling agents in C—N bond forming reactions [4]. Bertrand [15] and Singer [16] also reported copper NHC complexes bearing pyridine groups with copper in its +2 oxidation state (Figure 1).

Phenylboronic acids are effective cross-coupling agents for the generation of C—C, C—N, and C—heteroatom bonds [17]. Specifically, copper-mediated C—N bond-forming reactions using aryl boronic acids were developed independently by Chan, Evans, and Lam [4,18]. The Chan, Evans, and Lam (CEL) reaction uses an oxidative nucleophile-nucleophile approach using phenylboronic acid as the coupling agent with various amines (or other nucleophiles). The catalytic reactivity of this process is known to depend on various parameters, such as the reduction-oxidation properties of the copper catalyst, solvent, base, and temperature [19]. Aryl boronic acid activation can also lead to off-target side product generation, including protodeboronation to yield benzene derivatives, hydroxylation to form phenol, competitive addition of solvent (methanol) to form anisole, and homocoupling of two boronic acids to form diaryl compounds [20].

Here, we report the synthesis and characterization of a pincer copper(II)-NHC complex for aryl boronic acid activation toward generating C—O bonds. This copper(II) NHC complex shows strong σ donor and π-back bonding properties, which are promising attributes for development of catalysts for cross-coupling reactions in sustainable solvents [21,22]. This detailed study is focused on optimizing C—O bond generation from aryl boronic acids and solvent through a CEL-like mechanism, where the resulting phenols are well-used chemical intermediates having high economic value in chemical as well as in biological applications [23,24]. Phenol derivatives are often value-added intermediates in the preparation of many pharmaceutical and therapeutic molecules [25]. Due to their widespread utility, many efforts have been made to advance phenol preparation, including the conversion of phenylboronic acids to phenols [25,26]. Moreover, identifying conditions that favor C—O bond formation in water (and renewable solvents like methanol) also provide insight into conditions that limit C—O bond forming reactions. When targeting C—C and C—N bond forming processes in water, conditions can be selected that limit off-target (C—O bond forming) reactions and improve the efficiency of the C—C or C—heteroatom formation.

## 2. Materials and Methods

### 2.1. Materials

Picolyl chloride (TCI), benzimidazole, phenylboronic acid (ACROS organics, Bridgewater, NJ, USA), sodium carbonate, trisodium phosphate dodecahydrate (Fisher scientific, Waltham, MA, USA), Sodium dihydrogen phosphate monohydrate, potassium carbonate, disodium hydrogen phosphate heptahydrate (JT Baker, Deventer, The Netherlands), ammonium hexafluorophosphate, triethylamine, potassium hydroxide (Aldrich, St. Louis, MO, USA), copper(II) acetate (Alfa Aesar, Ward hill, MA, USA) were used as received. Perchloric acid, tetrabutylammonium hexafluorophosphate, and phosphorus(V) oxide were obtained from GFS chemicals, Inc. (Columbus, OH, USA), SACHEM, Inc. (Austin, TX, USA), and Sigma-Aldrich (St. Louis, MO, USA), respectively. All reagents used for this work were analytical grade and used as received. HPLC grade acetonitrile was obtained from Fisher Scientific (Waltham, MA, USA). The water used was initially deionized using a reverse osmosis system and further polished to be ~17 MΩ-cm.

### 2.2. Synthesis of 1,3-bis(pyridin-2-ylmethyl)-1H-benzo[d]imidazol-3-ium chloride (1)

A mixture of 2-picolyl chloride hydrochloride (5.93 g, 36.15 mmol), benzimidazole (4.25 g, 35.97 mmol), and sodium carbonate (4.55 g, 42.92 mmol) was mixed in 25 mL of ethanol in a 50 mL round bottom flask and refluxed for 36 h [27]. The solvent was removed completely under reduced pressure. The residue was redissolved in dichloromethane (DCM) (20 mL) and dried over CaSO_4_. This solution was filtered, and the DCM was removed under reduced pressure. The oily residue obtained was washed with THF (2 × 10 mL), giving dark brown solid (**1**). ^1^H NMR (CDCl_3_, 500 MHz): δ = 11.85 (s, 1H), 8.51 (d, *J* = 5.0 Hz, 2H), 7.90–7.85 (m, 2H), 7.84 (d, *J* = 8.0 Hz, 2H), 7.76 (td, *J* = 8.0 Hz, 1.5 Hz, 2H), 7.55–7.53 (m, 2H), 7.26–7.25 (m, 2H), 6.01 (s, 2H) ppm (Figure S1, see Supplementary Materials); ^13^C NMR (CDCl_3_, 125 MHz): δ = 152.4, 149.7, 144.1, 137.8, 131.7,127.1, 124.0, 123.8, 114.2, 52.7 ppm (Figure S2).

### 2.3. Synthesis of 1,3-bis(pyridin-2-ylmethyl)-1H-benzo[d]imidazol-3-ium hexafluorophosphate (2)

1,3-Bis(pyridin-2-ylmethyl)-1H-benzo[d]imidazol-3-ium chloride (0.168 g, 0.5 mmol) was dissolved in a minimum amount of H_2_O, followed by the addition of 5 equivalents of ammonium hexafluorophosphate (0.407 g, 2.5 mmol) [9]. A brown precipitate (**2**) was obtained and dried under vacuum. ^1^H NMR (CDCl_3_, 500 MHz): δ = 9.56 (s, 1H), 8.53 (d, *J* = 4.5 Hz, 2H), 7.86–7.84 (m, 2H), 7.78 (m, 2H), 7.61 (d, *J* = 7.8 Hz, 2H), 7.58 (m, 2H), 7.30 (m, 2H), 5.75 (s, 4H) (Figure S3); ^13^C NMR (CDCl_3_, 125 MHz): δ = 151.7, 149.9, 142.3, 138.0, 131.7, 127.4, 124.2, 123.4, 114.1, 52.7 ppm (Figure S4).

### 2.4. Synthesis of Copper(II)(1,3-bis(pyridin-2-ylmethyl)-1H-benzo[d]imidazol-3-ium) hexafluorophosphate (3)

1,3-Bis(pyridin-2-ylmethyl)-1H-benzo[d]imidazol-3-ium hexafluorophosphate (0.223 g, 0.5 mmol) was mixed with Cu(OAc)_2_ (0.0908 g, 0.5 mmol) in MeOH (3 mL) and stirred for 1 h at 50 °C. A blue precipitate of complex **3** was obtained which was isolated by filtration and washed with cold MeOH. This precipitate was dissolved in acetonitrile and MeOH and kept for slow evaporation. Blue-green crystals suitable for X-ray diffraction were obtained from this solution after approximately a week at room temperature. ESI-HRMS (m/z): observed 422.36 for [M—CH_3_OH2014PF_6_^−^]^+^; calcd 423.10 for [M—CH_3_OH—PF_6_^−^]^+^ (Figure S5); λ_max_/nm in CH_3_CN (ε_max_/M^−1^ cm^−1^): 555(165), 226(8440) and 259(9480).

### 2.5. General Procedure for the Copper-NHC-Catalyzed Formation of Phenol and Ethers

Reactions were carried out in a 25 mL round bottom flask charged with phenylboronic acid (0.1 mmol) and copper complex **3** (0.01 mmol) in 5 mL H_2_O or buffered aqueous solution. The reaction mixture was stirred at 70 °C for 6 h to yield phenol as the product. The reaction mixture was extracted in ethyl acetate and the % phenol conversion was obtained by GC-MS. Similar procedure has been followed for the conversion of phenylboronic acid to anisole and other ethers based on the different solvent media used. The % conversion of anisole and ethers formed were determined by using GC-MS by direct injection of the product mixtures. All reported % conversions represent the average value measured from 3 separate trials. Error and error bars reported here are one standard deviation of the mean recorded from these replications.

### 2.6. Experimental Methods

IR spectra were recorded using a Thermo Scientific Nicolet 6700 FT-IR. NMR spectra were obtained using a Bruker AVANCE III 500 MHz spectrometer at room temperature. ^1^H chemical shifts are reported vs. TMS and are referenced to the residual solvent peaks. Mass spectra were obtained using a Bruker UHPLC microTOF-Q II High Resolution MS system operating in ESI ionization mode. UV/vis spectra were obtained using an OLIS modernized HP 8452 UV/vis spectrophotometer. CV data were acquired with a CHI 620A (CH Instruments) electrochemical analyzer. The complex 2 solution was sparged with UHP Ar for 20 min to remove dissolved O_2_ and gas flow was maintained over the solution during CV acquisition. A blank CV was acquired prior the CV of the complex. The acetonitrile solution was scanned between −1.8 to 1.5 V vs. Ag/Ag^+^ reference electrode from the open circuit potential OCP, while that of HClO_4_ was scanned between −0.5 to 0.5 V vs. Ag/AgCl (Sat’d KCl). The aqueous CV potential is reported against normal hydrogen electrode (NHE) using the formula below:

$$\text{NHE}=\text{E}\left(\text{vs Ag}/\text{AgCl Sa}{\text{t}}^{\prime }\text{d KCl}\right)+0.199\text{V}$$

The X-ray intensity data were measured at low temperature (T = 100 K), using a three-circle goniometer platform with a fixed Kappa angle at = 54.74 deg Bruker AXS D8 Venture, equipped with a Photon 100 CMOS active pixel sensor detector. Monochromatized copper X-ray radiation (λ = 1.54178 Å) was selected for the measurement. The structure was solved in a centrosymmetric monoclinic unit cell; space group: P 1 2(1)/n 1, with Z = 4 for the formula unit, C_24_H_26_CuF_6_N_5_O_3_P. Crystal data and structure refinement details for complex **3** are shown in Figure S6 a,b and Table S6a–i. CCDC 2170331 for complex **3** contains the supplementary crystallographic data for this paper. The GC-MS was recorded in a Shimadzu QP-2010S GC-MS.

## 3. Results

**Scheme 1.**
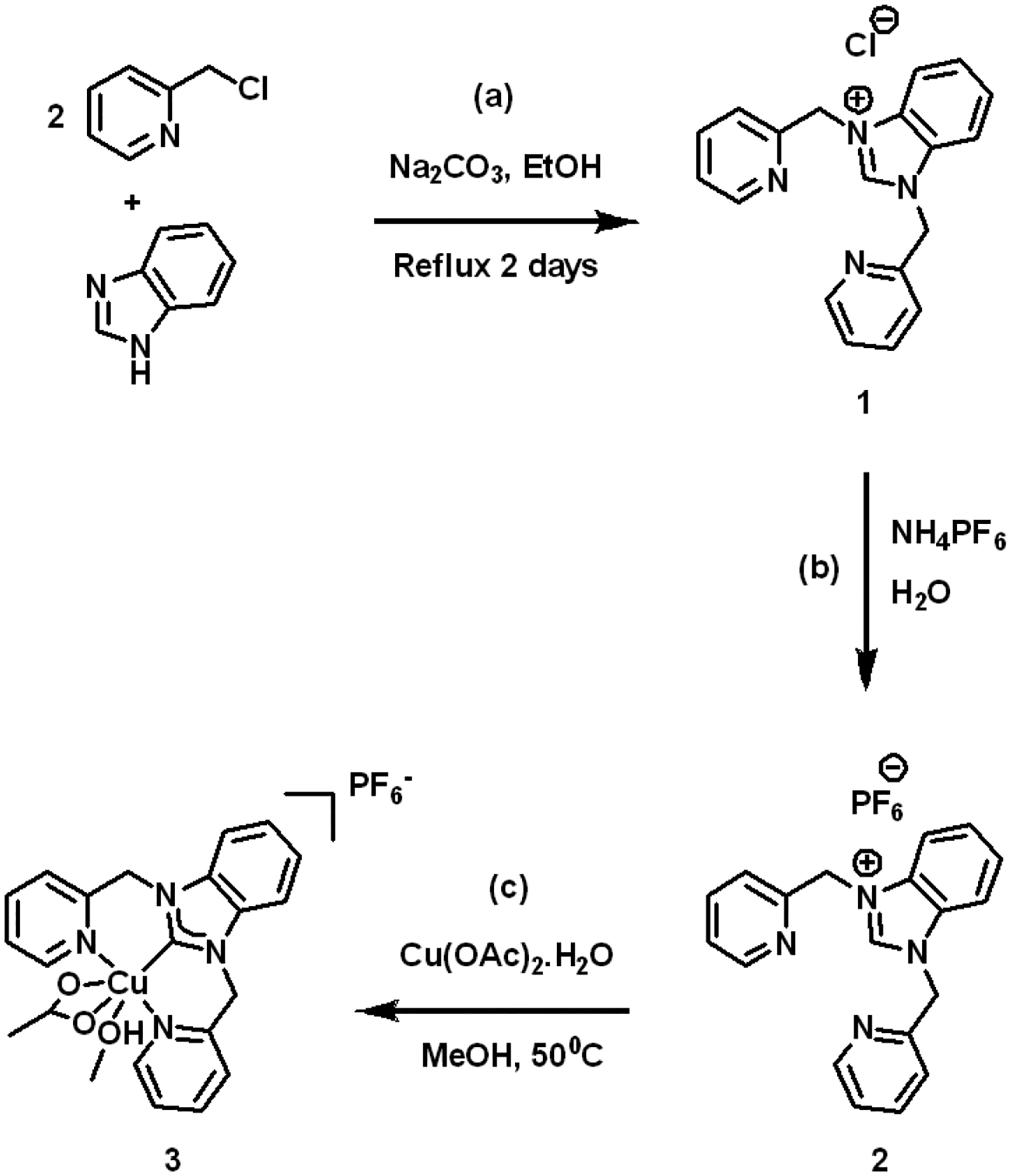
Reaction scheme for (**a**) ligand generation, (**b**) ion metathesis to PF_6_^−^ salt, and (**c**) carbene generation and metal ion ligation in the formation of the copper(II) NHC complex (**3**).

The synthesis of the tridentate copper-NHC complex was accomplished by initially synthesizing the tridentate NHC ligand in a two-step procedure as shown in Scheme 1a,b. The two-step methodology was initiated by reacting 2 equivalents of picolyl chloride with 1 equivalent of benzimidazole and this solution was heated to reflux in EtOH for 48 h. This reaction generated the tridentate ligand chloride salt (**1**) in a 68% yield. The chloride counterion was exchanged with PF_6_^−^ forming compound **2**.

Compound **2** and Cu(OAc)_2_ were dissolved in MeOH and heated to 50 °C for 1 h. A dark blue precipitate of complex **3** was obtained with a 75% yield (Scheme 1c). The precipitate was collected and characterized by X-ray crystallography, cyclic voltammetry, and combination of other spectroscopic techniques including UV-Vis and FT-IR. Finally, **3** was also characterized by HR-MS.

**Figure 2.**
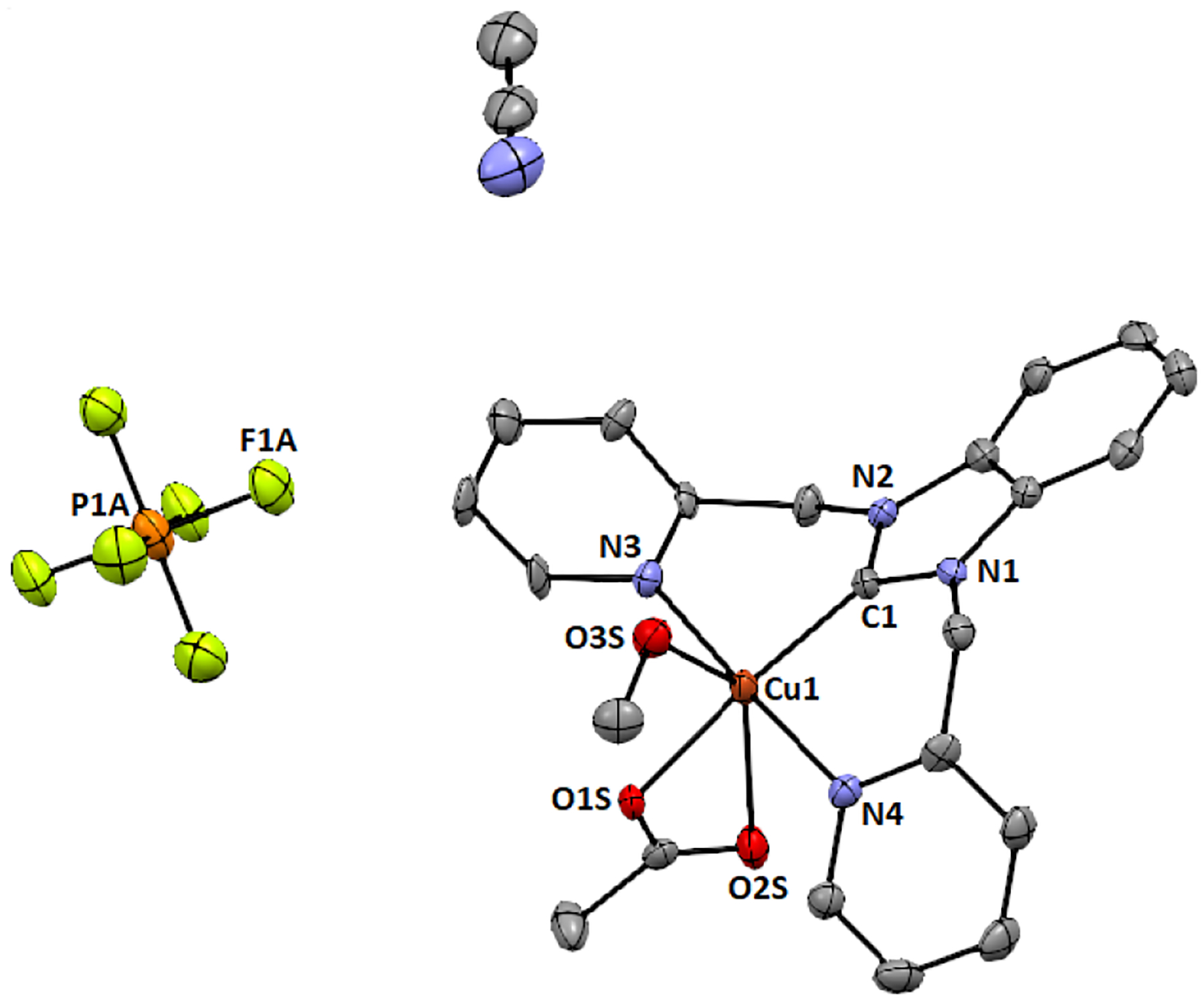
ORTEP representation of complex **3**. Ellipsoids are drawn at 50% probability and hydrogen atoms are removed for clarity. Selected bond lengths (Å): Cu1-C1 = 1.935(8); Cu1-N4 = 2.112(7); Cu1-N3 = 2.066(7); Cu1-O3S = 2.287(6); Cu1-O1S = 1.975(5); Cu1-O2S = 2.758. Angles (°): N3-Cu1-N4 = 177.2(3); O1S-Cu1-C1= 162.1(3); N3-Cu1-C1 = 87.7(3); N3-Cu1-O3S = 90.9(2); N3-Cu1-O2S = 94.40.

A single crystal of **3** was generated from slow solvent evaporation from a mixture of acetonitrile and methanol. The X-ray crystal structure of complex **3** is shown in Figure 2.

The crystal structure of the complex reveals that the crystallographic asymmetric unit of **3** contains one complex cation and one hexafluorophosphate anion. The copper(II) metal center is hexacoordinated by the two nitrogen and one carbon of the tridentate ligand and three oxygen atoms of the methanol and acetate units. The two nitrogen atoms (Cu1—N3 = 2.066 Å and Cu1—N4 = 2.112 Å) and one carbon atom (Cu1—C1 = 1.935 Å) of the tridentate NHC ligand and one oxygen atom (Cu1—O1S = 1.975 Å) of the acetate moiety occupies the equatorial square planar position of the octahedron while the other oxygen atoms of the methanol (Cu1—O3S = 2.287 Å) and acetate (Cu1—O2S = 2.758 Å) moiety occupy the longer axial positions (Table S6 i). This axial elongation is common in copper(II) complexes through the Jahn-Teller distortion, but also highlights the labile nature of these O donating ligands. The T = R_in_/R_out_ = 0.8015 value (where, R_in_ = (1.935 + 1.975 + 2.066 + 2.112 Å) /4 and R_out_ = (2.758 + 2.287 Å)/2) suggests that the complex is tetragonally distorted octahedral [28–31]. This distortion in the octahedral structure was further confirmed by the sum of the bond angles 339.30° around copper(II) (∠N3—Cu1—N4 is 177.20(3)° and ∠C1–Cu1–O1 is 162.10(3)°), revealing that the equatorial plane around copper(II) in **3** is not perfectly square planar (Table S6 i). One aspect that requires additional consideration is the Cu—C bond and its role in stabilizing this complex. The relatively short Cu—C bond distance (1.935 Å) is consistent with strong σ donation and possibly π back bonding, which may be attributable to the high stability of this complex. The powder XRD peaks are consistent with the simulated powder data generated from the single crystal X-ray diffraction data (Figure S7a–c).

**Figure 3.**
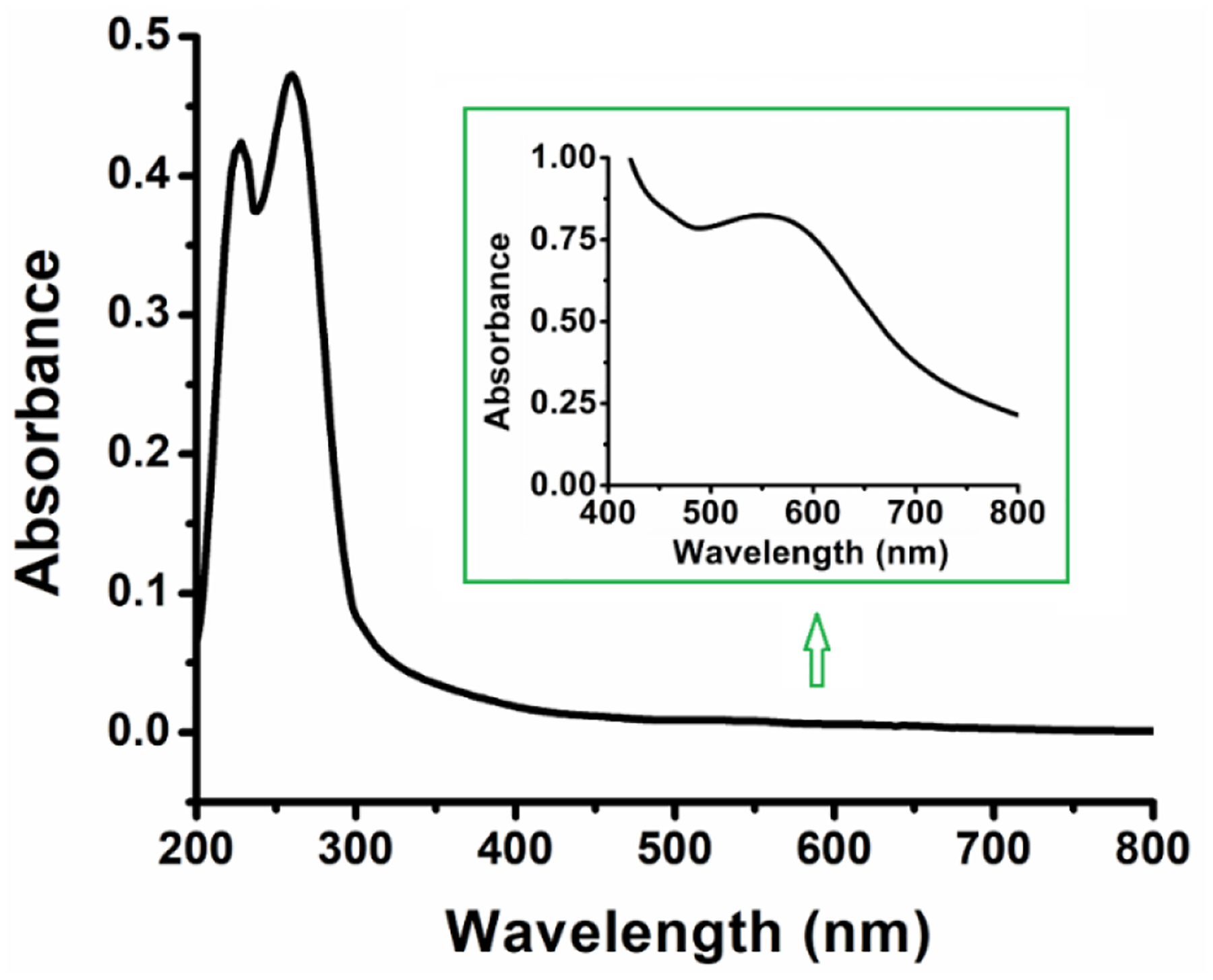
UV–Vis absorption spectra of complex **3** in CH_3_CN. (conc: 5 × 10^−5^ M; Inset conc: 5 × 10^−3^ M).

The UV-Vis for the complex **3** was taken in acetonitrile and it shows a d-d transition (λ_max_ = 555 nm; ε_555_ 165 M^−1^ cm^−1^) at significantly higher energy than Cu(OAc)_2_, which is consistent with the strong field nature of the NHC ligand stabilizing the t_2g_ orbitals in this complex. This observation is consistent with the tetradentate copper NHC complex with 2 carbene donor ligands synthesized previously in our group (λ_max_ = 505 nm; ε_505_ = 333 M^−1^cm^−1^) [4]. The complex also exhibits two absorption band at 226 nm (ε_226_ = 8,440 M^−1^cm^−1^) and 259 nm (ε_259_ = 9,480 M^−1^cm^−1^) attributable to the ligand π → π* or n → π* transitions in the aromatic regions of this complex (Figure 3).

**Figure 4.**
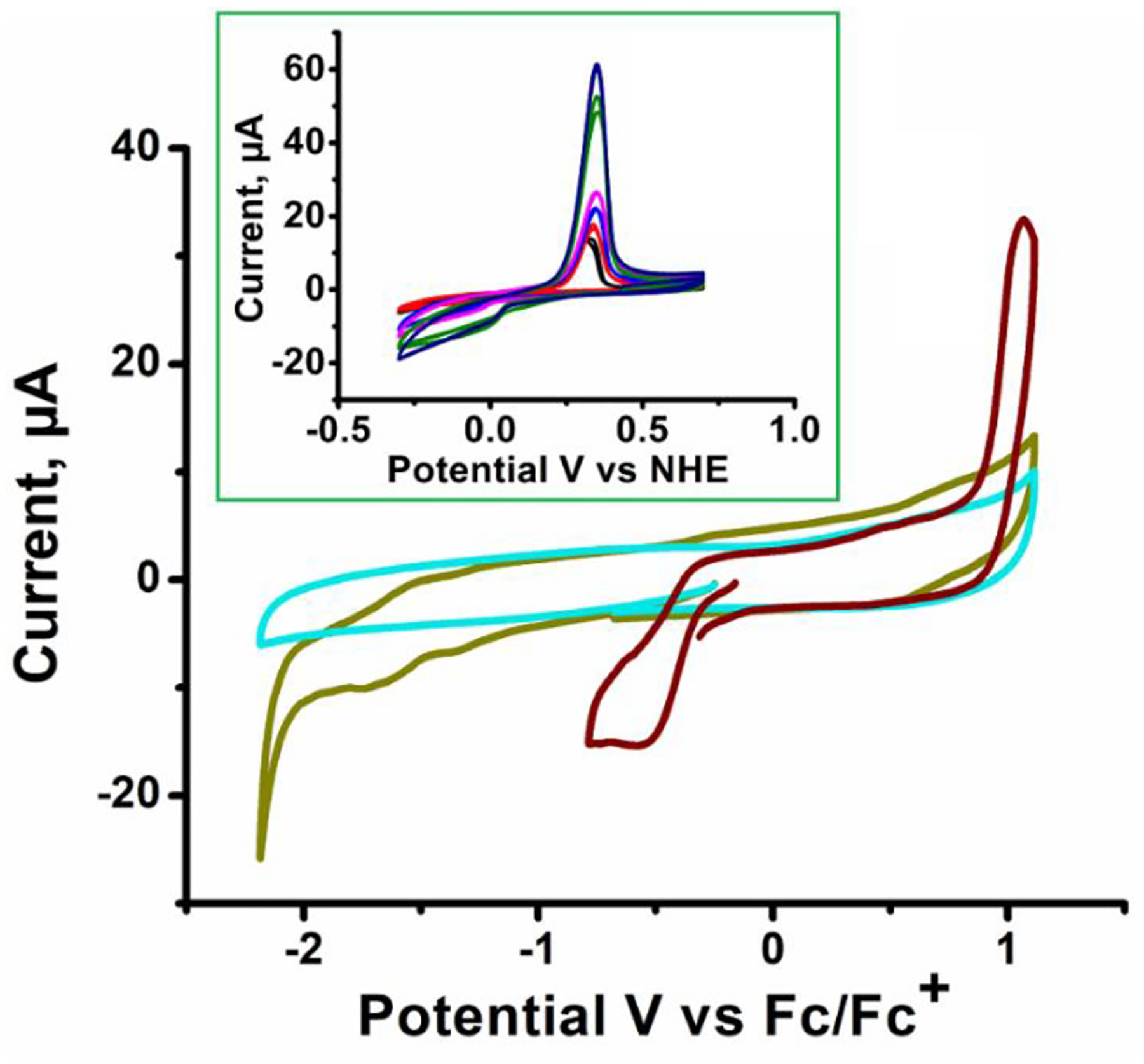
Cyclic voltammograms of 1 mM blank (turquoise), ligand (green) and complex **3** (brown) in 0.1 M [NBu_4_][PF_6_]/CH_3_CN. Scan rate at 100 mV/s. (Inset) Cyclic voltammograms complex **3** in 0.1 M HClO_4_ at varying scan rate for complex **3** in 0.1 M HClO_4_. Scan rates (mV/s): 10 (black), 20 (red), 50 (blue), 75 (pink), 100 (green), 150 (purple).

The cyclic voltammogram of complex **3** is shown in Figure 4. The CV of the ligand (**2**) showed two irreversible reduction peaks at −1.75 and −1.365 V and an oxidation peak at 0.700 V. The copper(II) complex (**3**) showed a shift in the reduction peak in the potential range from 0 to −0.785 V vs. Fc^+^/Fc [4,32,33]. Complex **3** exhibits an intense irreversible oxidation peak at E_pa_ = 1.069 V in comparison with the oxidation peak found in the ligand, and thus there is a distinctive anodic shift in the oxidation peak seen in the copper(II) complex. This single oxidation peak is consistent with a ligand-based oxidative event [34,36].

The CV of **3** in 0.1 M HClO_4_ solution is presented in Figure 4. Both a cathodic and an anodic peak were observed for the complex when scanned between −0.5 to 0.5V vs. Ag/AgCl [34]. The irreversible cathodic reduction peak at −0.108 V vs. NHE is likely due to a 2 e- Cu^2+^ to Cu reduction, while the sharp anodic oxidation peak at 0.325 V is consistent with a stripping peak for the oxidation of deposited Cu to solution Cu^+^ [35–37]. Significant fouling of the GCE electrode surface was noted during scanning requiring the electrode to be polished between scans. The fouling and stripping wave suggest decomposition of the complex during reduction in HClO_4._

The IR spectra of **1**, **2**, and **3** were recorded (c.f. Figure S8). These spectra are generally considered to be qualitative spectroscopic characterization data for each of the ligand and complex generated without specific isotopic assignments. However, vibrational modes that are consistent with C=N bonds in **1** and **2** are observed at 1592 and 1594 cm^−1^, respectively. A distinctive shift in this region is observed upon coordination of copper(II) of this tentative C=N vibrational mode to 1615 cm^−1^ for complex **3** consistent with the coordination of copper to the NHC ligand as shown in Figure S8 [38].

Complex **3** was used as a catalyst for aryl boronic acid activation and C—O bond formation. Specifically, our initial efforts were focused on generating phenol from phenylboronic acid under the following conditions: 0.1 mmol phenyl boronic acid and 10 mol% of complex **3** in 5 mL water at 50 °C under aerobic conditions. Samples from this reaction were assessed after 2 h to quantify phenol generation as an indicator of % conversion of this process. The reaction was screened with differing amount of catalyst, reaction times, temperature, solvents, and bases. The formation of phenol was determined by GC-MS. Phenol was used to generate a calibration response curve associated with an optimized separation method, and this peak assignment was validated using the corresponding mass spectra.

**Figure 5.**
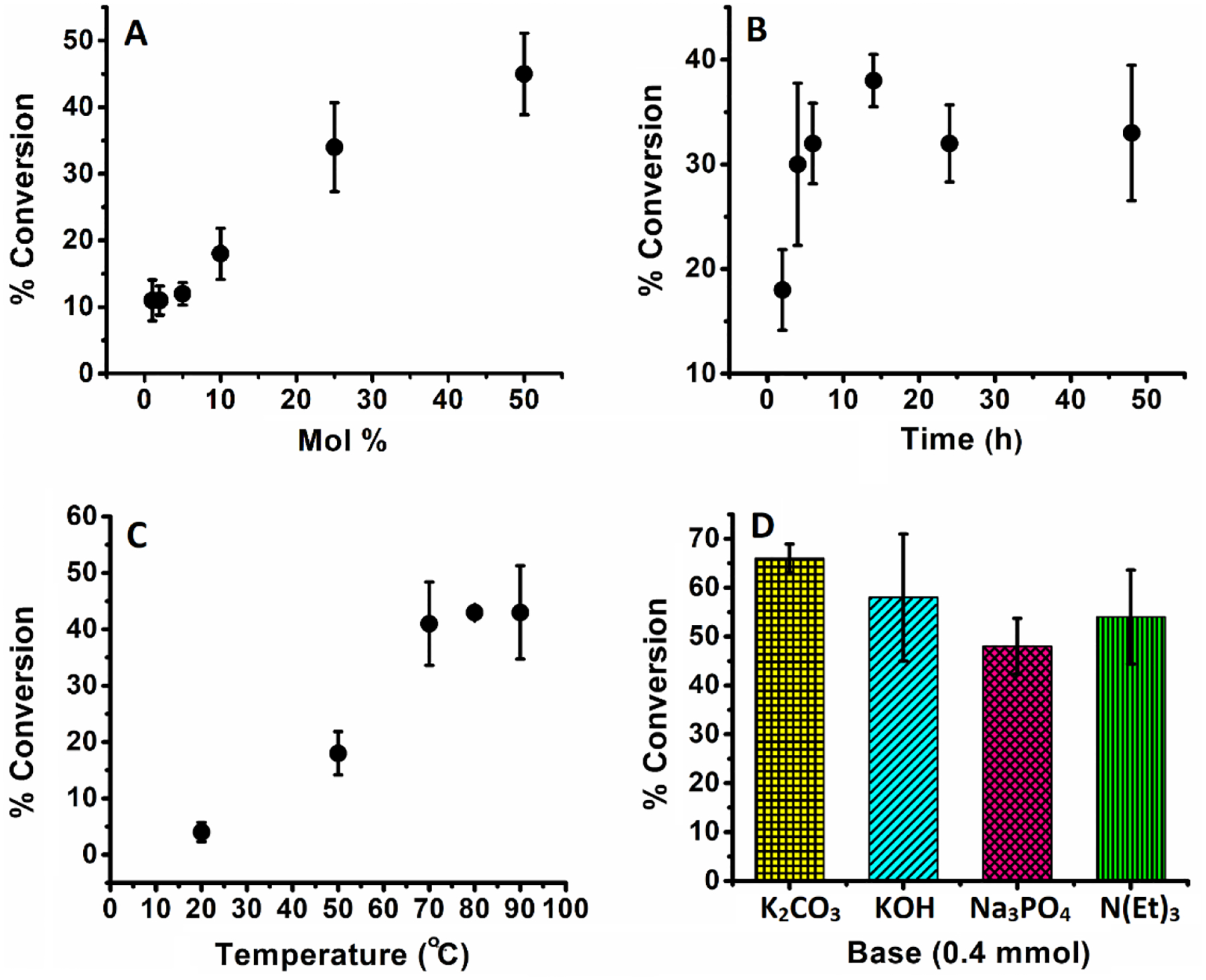
(**A**) Effect of amount of catalyst in hydroxylation of phenyl boronic acid to phenol. Reaction conditions: phenyl boronic acid (0.1 mmol), H_2_O (5 mL), 50 °C, 2 h. (**B**) Effect of reaction time in hydroxylation of phenyl boronic acid to phenol. Reaction conditions: phenyl boronic acid (0.1 mmol), catalyst (10 mol%), H_2_O (5mL), 50 °C. (**C**) Effect of reaction temperature in hydroxylation of phenyl boronic acid to phenol. Reaction conditions: phenyl boronic acid (0.1 mmol), catalyst (10 mol%), H_2_O (5 mL), 6 h. (**D**) Effect of different bases in hydroxylation of phenyl boronic acid to phenol. Reaction conditions: phenyl boronic acid (0.1 mmol), catalyst (10 mol%), H_2_O (5 mL), 70 °C, 6 h.

Six different catalyst concentrations with respect to the substrate were screened under otherwise identical reaction conditions as shown in Table 1 (entries 1–6). Phenol concentrations were measured in these trials after 2 h and correlated with % conversion based on initial phenyl boronic acid concentration. The observed % conversion for this process was consistent (11–12%) when the concentration of the catalyst was changed from 1 to 5 mol%. Upon increasing the concentration of the catalyst to 10 mol% there was a slight increase in % conversion to ~18%. At higher catalyst concentrations, the % conversion reached to 34% and 45% for 25 and 50 mol% catalyst, respectively. When plotted this data has a pseudo-linear relationship between % conversion and mol % catalyst (Figure 5A), which is consistent with a pseudo-first-order dependance on catalyst loading. Keeping this catalyst loading data under consideration, 10 mol% catalyst loading was for further optimization. A control experiment conducted in the absence of copper(II) does not activate phenylboronic acid to phenol. Furthermore, the catalytic activity of Cu(OAc)_2_ was compared to that measured for **3**. Complex **3** supports almost twice the conversion of Cu(OAc)_2_ under analogous reaction conditions.

Phenol generation was monitored at different times; a steep increase to 30% was observed after 2 h of reaction time, as shown in Table 1 (entries 4, 7–11). Increasing the reaction time beyond 2 h slowly increased phenol production up 38% conversion at 14 h. When time is plotted against % conversion there is clearly limited changes to the % conversion beyond 6 h (Figure 5B). For convenience, the 6 h reaction time giving a phenol conversion of 32% was selected to proceed further to check the effect of temperature on the phenol conversion. The effect of temperature influenced the % conversion. A 4% conversion was observed at room temperature. At 50 °C, the % conversion increased to 18%. At 70 °C the % conversion was 41%. Increasing the temperature beyond 70 °C resulted in no further significant change in % conversion (Table 1 (entries 8, 12–15) and Figure 5C).

**Figure 6.**
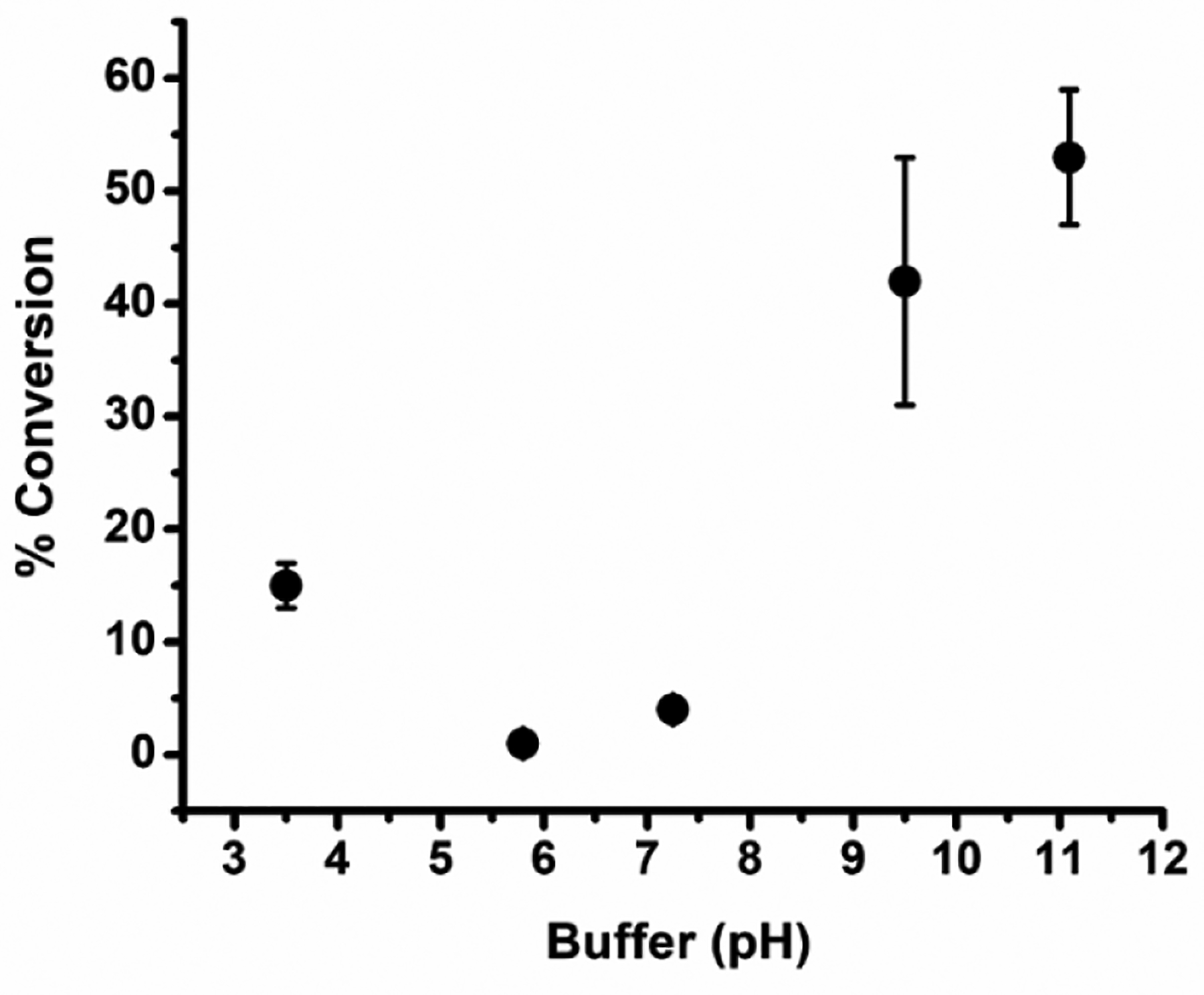
Effect of buffers in hydroxylation of phenyl boronic acid to phenol. Reaction conditions: phenyl boronic acid (0.1 mmol), catalyst (10 mol%), buffer (5 mL of 20 mM carbonate pH 11.1 and 9.5, 20 mM phosphate pH 5.8 and 7.3, and 20 mM acetate buffer pH 3.5), 6 h, 50 °C.

Traditionally, there is a major impact on metal catalyzed aryl boronic acid activation pathways with the addition of base. Here we also investigated the influence on phenol generation by addition of some common inorganic and organic bases. Initially, we explored the effect of potassium carbonate, where 1 equivalent of K_2_CO_3_ increased the % conversion to 53%. Adding additional equivalents of K_2_CO_3_ (4 equivalents) supported 63% reaction conversion. However, when other bases were screened, including KOH, Na_3_PO_4_, and triethylamine, no improvement in phenol generation was observed (Table 1 (entries 16–20) and Figure 5D. Notwithstanding the superior activity of the catalyst examined in various bases, we also investigated its action in different buffer solutions. In a buffer cocktail containing carbonate, phosphate, and acetate buffers between pH 3.5 to 11, it was observed that at low pH (i.e., pH 3.5) the % conversion reached 15%. At neutral pH values (i.e., pH 5.8–7.25), the conversion of phenylboronic acid to phenol gave minimal yields (~4%). At high pH (pH 9.5–11), the % conversion increased significantly to 42 and 53%, respectively (Figure 6).

The phenylboronic acid activation was also attempted in a series of polar protic solvents, including methanol, ethanol, phenol, and *t*-butanol, to generate the corresponding ether products under similar optimized reaction conditions (10 mol% catalyst, 70 °C). A 50% conversion of phenylboronic acid to anisole was observed in methanol. Other targeted ethers were only generated in trace amounts, including ethoxybenzene, *t*-butoxybenzene, and diphenyl ether, from the activation of phenylboronic acid in ethanol, *t*-butanol, and phenol, respectively.

Additionally, we also screened several common catalytic systems based on copper metal and ligands such as 2,2′-bipyridine (bipy) and 1,10-phenanthroline (phen) to produce phenol under the identical optimized reaction conditions (Table 2). It was observed that the conversion could only reach up to 37% at a catalyst loading of 10 mol % as shown in Table 2.

Finally, we performed a competition study activating phenylboronic acids in water containing a more potent nucleophile, namely imidazole. This study pits water against imidazole as the target for arylation, much like the C—N bond forming reactions commonly targeted using CEL-type coupling reactions. When all reaction parameters are kept similar to those of Entry 17 in Table 1, except for the addition of 1 equivalent of imidazole to the reaction mixture, phenol generation is increased to 71% conversion of the phenylboronic acid to phenol. However, on increasing the concentration of imidazole to 20 equivalents, the % conversion dropped to 31%. On further increasing it to 50 equivalents showed only 2% of phenol generation. No phenol generation was observed on further increasing the concentration of imidazole beyond 50 equivalents.

## 4. Discussion

In the present study, we report a facile synthesis of the tridentate copper-NHC catalyst, based on a benzimidazole-based carbene donor with two pendant pyridyl N donor groups. The complex is similar the tridentate copper(II)-NHC reported by Singer [16], and is a 3-coordinate derivative of a tetradentate copper-NHC developed by our group [4]. The visible absorption for complex 3 shows a broad d-to-d transition band (λ_max_ = 555 nm; ε_555_ = 165 M^−1^cm^−1^) that is lower in energy when compared to that of the tetradentate copper-NHC complex synthesized previously (λ_max_ = 505 nm; ε_505_ = 333 M^−1^cm^−1^) [4]. Interestingly, the X-ray structure obtained shows that the Cu1—C1 bond distance of 1.935 Å in complex 3, which is slightly shorter than the 1.956 and 1.962 Å bond lengths seen in similar Cu—C(carbene) bonds in the tetradentate copper-NHC complex reported from our group earlier [4]. We attribute this short bond to the increased covalent character of this interaction compared to normal dative bonds. This observation is similar to other copper(II)—C(carbene) distances in copper-NHC complexes [39]. This is evident from Singer’s single-crystal X-ray diffraction study of the bis(pyridine)−NHC−copper(II) complexes [(CN_2_)Cu-(MeOH)_2_(SiF_6_)] and [(CN_2_)Cu(Cl)(SO(CD_3_)_2_)]X; the copper(II)−C(carbene) distances 1.915(3) and 1.932(2) Å, respectively, fall at the short end of those of known copper(II) NHC complexes (Table 3) [16]. Owing to the geometric constraints of the NCN pincer ligand, shorter copper(II)−C(carbene) distances are common in such copper carbene complexes. Consequently, the copper(II)−C(carbene) bond length in Long’s copper(II) complex [(PY4Im)Cu(MeCN)]^2+^ (1.889(4) Å) is even much shorter, although the copper center is clearly six-coordinate (Table 3) [40].

In terms of the phenol generation observed here, catalyst loading plays a key role in reactivity; catalyst loading is proportional to phenol generation. Generally, phenol generation occurs within the first hours of a reaction, where additional time does not dramatically influence product production. Phenol production is highly dependent on the reaction temperature, where increasing the temperature increases the % conversion considerably. The highest phenol production was observed at 70 °C or higher. It was also observed that the addition of base supports phenol generation. The pH of the reaction showed the best yields under basic conditions, which is consistent with observations regarding adding proton acceptors to the reaction medium. Further analysis showed that phenol generation was enhanced under both acidic and basic conditions, and at neutral pH only a trace amount of phenol is generated. Under acidic conditions, some aspect of our catalyst could potentially be protonated, increasing the lability of one of the copper ligands. At acidic pH values, which enhanced the phenol generation, it seems that protonation of the acetate ion could perhaps decrease the Coulombic attraction between the Cu^2+^ and acetate ions, making it easier to displace acetate with either water or a phenyl unit needed for a productive reaction.

**Scheme 2.**
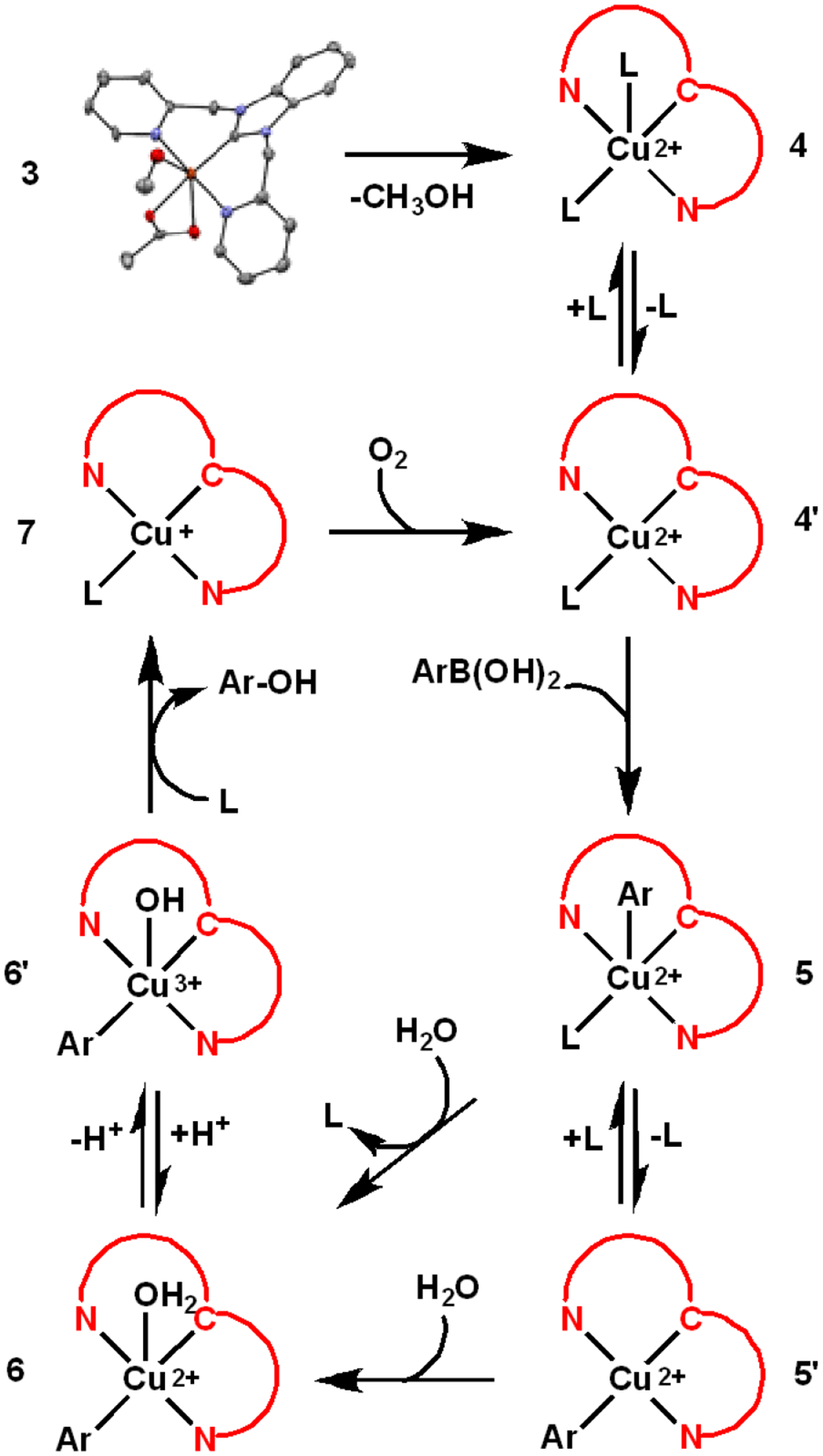
A plausible mechanism for phenol formation from phenylboronic acid catalyzed by complex **3**.

Basic conditions effectively increase the hydroxide concentration, which leads to higher phenol generation. This is likely due to the deprotonation of a copper(II)-bound water molecule, which increases the nucleophilicity of the Lewis acid/copper-activated water moiety, allowing it to better react with the activated phenyl unit. Complex 3 can also undergo transitions to give 4- and 5-coordinated species during the reaction in solution due to the hemilability of the pyridine coordination modes. A reaction mechanism for this process can be postulated (Scheme 2) that follows a similar course to that of other CEL coupling reaction pathways that have been well vetted in the literature [4,19]. Due to the weak interaction of the acetate to the copper center (Cu1-O2S = 2.758 Å), one of the bonds breaks to form a 4-coordinated species (4′). This 4-coordinate species activates the aryl boronic acid leading to a metal bound aryl moiety (5); this activated species undergoes a ligand substitution directly (6) or through a 4-coordinate intermediate (5′), and allows one water molecule to coordinate at the coordination site (6) [19]. Followed by a deprotonation, the potential of copper(II) can shift forming an oxidized copper(III) species (6′). This complex can undergo reductive elimination to give phenol as a product and generate a copper(I) species (7), which can reoxidize to the initiation point, copper(II), by a molecular oxygen (4′). Further investigation is needed to test this proposal. However, it is interesting to note that under competitive conditions in which both water and imidazole are present, the C—N bond-forming reaction is the favored process. When 50 equivalents of imidazole are added to our optimized reaction conditions, only 2% phenol conversion is noted. Under these conditions we estimate that water is 5500 times more abundant than imidazole, yet almost all the aryl boronic acids are utilized for C—N bond formation.

## 5. Conclusions

Together, these catalytic observations give a clear indication of the conditions for generating phenol from the activation of arylboronic acids using the reported copper(II) NHC complex in water. This C—O bond forming reaction has been shown to have significant merit on its own [23,41–46], but it also provides insight into conditions where phenol generation is limited. C—O bond formation occurs in a similar pathway to other CEL bond forming processes but requires higher energy conditions (high temperature) and carefully controlled pH. When the phenol generation process is challenged by adding additional nucleophilic potential substrates, we see that even at low concentrations of imidazole, this C—O formation process is almost completed replaced by C—N bond formation. This indicates that the nucleophilicity of the substrate dramatically governs the rate of reaction, where 20- to 50-equivalents of imidazole out compete ~55 M water. Careful direction of phenylboronic acid reaction processes can be achieved by carefully controlling (1) pH, (2) temperature, and (3) nucleophile concentration. Using these experimental handles, this reaction can be directed to solvent-focused C—O bond formation or toward other C—heteroatom bond-forming reactions.

## Supplementary Material

Supplemental Materials

## Figures and Tables

**Table 1.** Optimization of reaction conditions for complex **3** catalyzed formation of phenol from phenylboronic acid^a^.

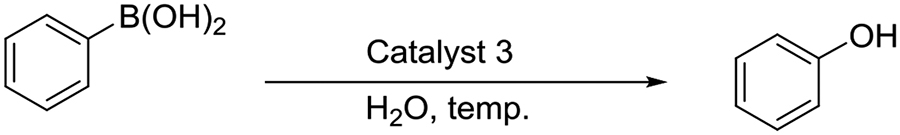						
Entry	Catalyst Loading (Mol%)	Time (h)	Temperature (°C)	% Conversion	TON^b^	Base (equivalents)
1	1	2	50	11	11	-
2	2	2	50	11	5.5	-
3	5	2	50	12	2.4	-
4	10	2	50	18	1.8	-
5	25	2	50	34	1.4	-
6	50	2	50	45	0.9	-
7	10	4	50	30	3	-
8	10	6	50	32	3.2	-
9	10	14	50	38	3.8	-
10	10	24	50	32	3.2	-
11	10	48	50	33	3.3	-
12	10	6	RT	4	0.4	-
13	10	6	70	41	4.1	-
14	10	6	80	43	4.3	-
15	10	6	90	43	4.3	-
16	10	6	70	53	5.3	K_2_CO_3_ (1)
17	10	6	70	66	6.6	K_2_CO_3_ (4)
18	10	6	70	58	5.8	KOH (4)
19	10	6	70	48	4.8	Na_3_PO_4_ (4)
20	10	6	70	54	5.4	NEt_3_ (4)

a Reactions were carried out with 0.1 mmol of the substrate phenylboronic acid and 5 mL of solvent (H_2_O).

b TON (turnover number) = moles of the product per mole of the catalyst.

**Table 2.** Optimization of reaction conditions for some common catalyst systems for the formation of phenol from phenylboronic acid^a^.

	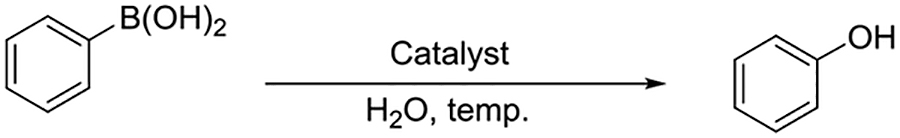			
Entry	Catalyst	Catalyst loading (mol %)	% Conversion	Base (Equivalents)
1	CuCl_2_	10	31	-
2	CuCl_2_ + Bpy (1:1)	10	36	-
3	CuCl_2_ + Bpy (1:2)	10	32	-
4	CuCl_2_ + Phen (1:1)	10	34	-
5	CuCl_2_ + Phen (1:2)	10	37	-

a Reactions were carried out with 0.1 mmol of the substrate phenylboronic acid and 5 mL of solvent (H_2_O) at 70 °C for 6 h.

**Table 3.** Copper(II)—C(carbene) bond distances in some copper-NHC complexes.

Entry	Structure	Copper(II)—C bond Distances (Å)	Ref.
1	**3**	1.935(8)	herein
2	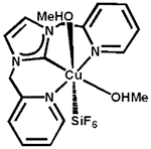	1.915(3)	[16]
3	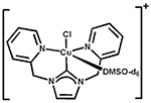	1.932(2)	[16]
4	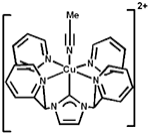	1.889(4)	[40]

## Data Availability

All data is available upon request.
