# Supplementary material for: Copper(II) NHC Catalyst for the Formation of Phenol from Arylboronic Acid
Source: Chemistry (Basel). Author manuscript; Available in PMC 2023 Nov 29. (PMC10686634; doi:10.3390/chemistry4020040)
Supplement: Supplemental Materials [file NIHMS1894793-supplement-Supplemental_Materials.docx]

**Supplementary Materials**

**Copper(II) NHC catalyst for the formation of phenol from arylboronic acid**

Mitu Sharma, Bhupendra Adhikari, Raymond Femi Awoyemi, Amanda M. Perkins, Alison K. Duckworth, Bruno Donnadieu, David O Wipf, Sean L. Stokes, Joseph P. Emerson^*,1^

*^1^Department of Chemistry, Mississippi State University, Mississippi State, MS 39762-9573*

** Correspondence: JEmerson@chemistry.msstate.edu; Tel.: 1.662.325.4633*

**Figure S1** ^1^H NMR of compound **1**.

**Figure S2** ^13^C NMR of compound **1**.


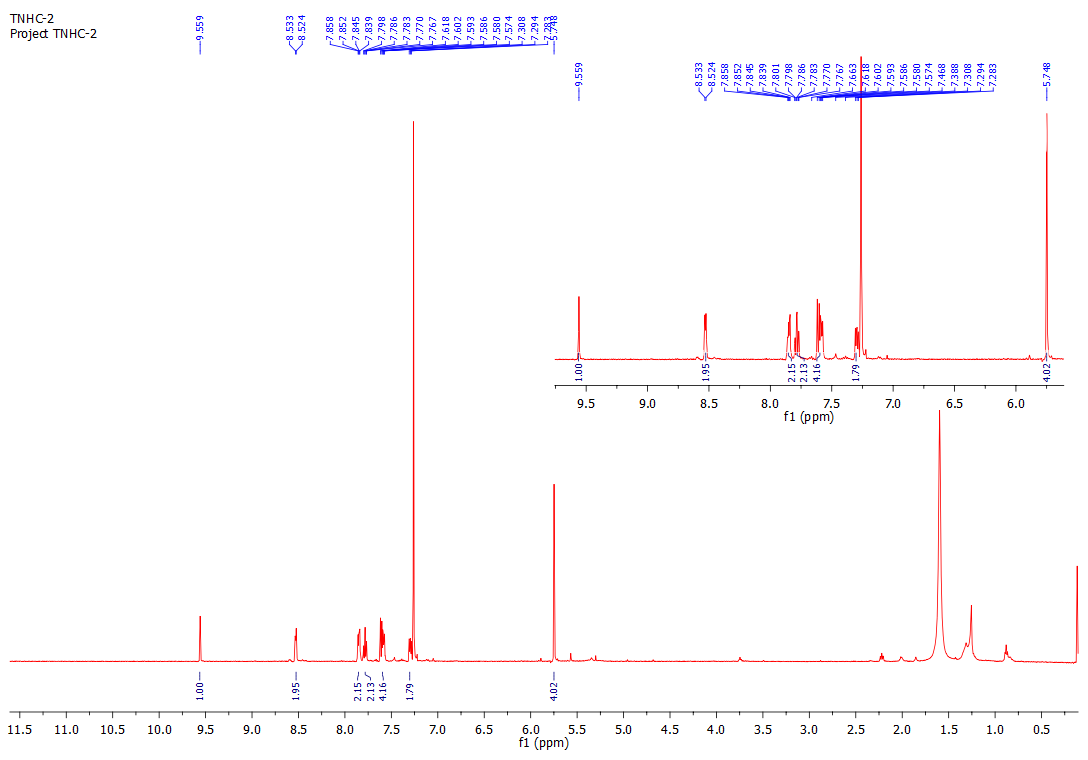


**Figure S3** ^1^H NMR of compound **2**.

**Figure S4** ^13^C NMR of compound **2**.


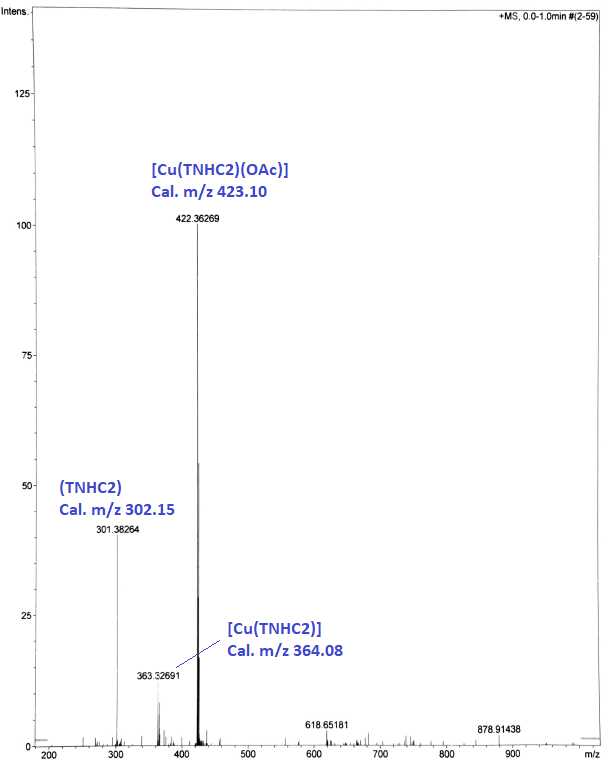


**Figure S5** HR-MS of complex **3** in CH_3_CN solution (TNHC2= tridentate ligand **2** moiety).

**Crystal Structure Report for complex 3**

A dark green prism single crystal of C_24_H_26_CuF_6_N_5_O_3_P, approximate dimensions (0.080x 0.142 x 0.182) mm^3^, was selected for the X-ray crystallographic analysis and mounted on a cryoloop using an oil cryoprotectant (Figure S6 a). The X-ray intensity data was measured at low temperature (T = 100K), using a three circles goniometer Kappa geometry with a fixed Kappa angle at = 54.74 deg Bruker AXS D8 Venture, equipped with a Photon 100 CMOS active pixel sensor detector. A monochromatized Cu X-ray radiation (λ = 1.54178 Å) was selected for the measurement. All frames were integrated with the aid of the Bruker SAINT software **^1^** using a narrow-frame algorithm. The integration of the data using a monoclinic unit cell yielded a total of 30831 reflections to a maximum θ angle of 68.25° (0.83 Å resolution), of which 4842 were independent (average redundancy 6.367, completeness = 99.1%, R_int_ = 25.54%, R_sig_ = 11.74%) and 3138 (64.81%) were greater than 2σ (F^2^). The final cell constants of a = 6.9851(3) Å, b = 26.4631(10) Å, c = 14.4471(6) Å, β = 94.430(3) °, volume = 2662.53(19) Å^3^, are based upon the refinement of the XYZ-centroids of 1380 reflections above 20 σ (I) with 6.680° < 2θ < 149.0°. Data were corrected for absorption effects using the Multi-Scan method: (SADABS) **^2^**. The ratio of minimum to maximum apparent transmission was 0.788. The calculated minimum and maximum transmission coefficients (based on crystal size) are 0.6640 and 0.8280. The structure was solved in a monoclinic unit cell; Space group: P 1 2(1)/n 1, with Z = 4 for the formula unit, C_24_H_26_CuF_6_N_5_O_3_P (Figure S6 b). Using the Bruker SHELXT Software Package **^3^**, refinement of the structure was carried out by least squares procedures on weighted F^2^ values using the SHELXTL-2018/3 **^4^** included in the APEX4 v2021, 4.0, AXS Bruker program **^5^**. Hydrogen atoms were localized on difference Fourier maps but then introduced in the refinement as fixed contributors in idealized geometry with an isotropic thermal parameters fixed at 20 % higher than those carbons atoms they were connected. The PF_6_^-^ anion was found statistically disordered on two positions and anisotropically refined with a ratio of occupancy equal to: 50%. A molecule of acetate and methanol were localized coordinated on the metal center and a free molecule of acetonitrile. The final anisotropic full-matrix least-squares refinement on F^2^ with 429 variables converged at R1 = 10.72%, for the observed data and wR2 = 21.98% for all data. The goodness-of-fit: GOF was 1.132. The largest peak in the final difference electron density synthesis was 0.590 e^-^/Å^3^ and the largest hole was -0.669 e^-^/Å^3^ with an RMS deviation of 0.131 e^-^/Å^3^. Based on the final model, the calculated density was 1.599 g/cm^3^ and F (000), 1308 e^-^. Graphics were performed using softwares: Mercury V.4.2.0: (<https://www.ccdc.cam.ac.uk/>) and POV-Ray v 3.7: (The Persistence of Vision Raytracer, high quality, Free Software tool).

***References associated with crystallographic analysis:***

**1** Saint Program included in the package software: APEX4 v2021.4.0. Brukersupport.com 05/06/2021

**2** Bruker (2001).*Program name.*Bruker AXS Inc., Madison, Wisconsin, USA. Brukersupport.com 05/06/2021

**3** SHELXT-Integrated space-group and crystal-structure determination Sheldrick, G. M. Acta Crystallogr., Sect. A 2015, **A71**, 3-8.

**4** SHELXTL Sheldrick, G. M. Ver. 2018/3. Acta Crystallographica. Sect C Structural Chemistry **71**, 3 - 8.

**5** APEX4 v2021, 4.0, AXS Bruker program. Brukersupport.com 05/06/2021

| 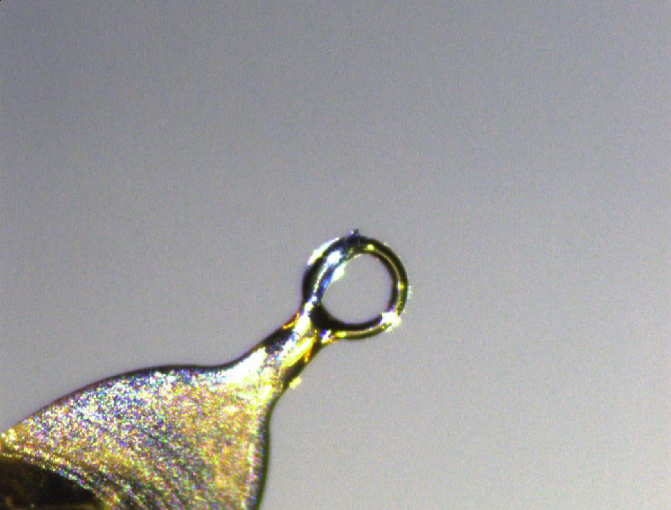 | 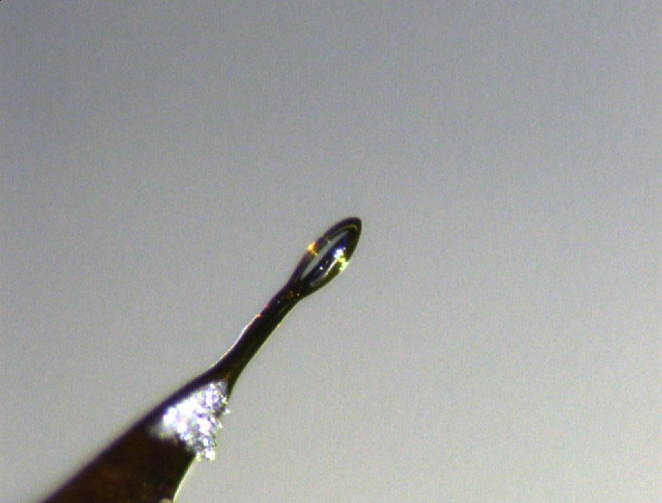 |
| --- | --- |
| 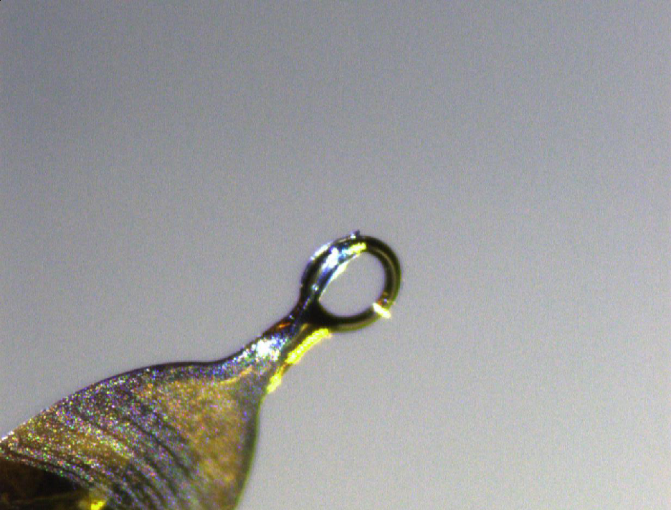 | 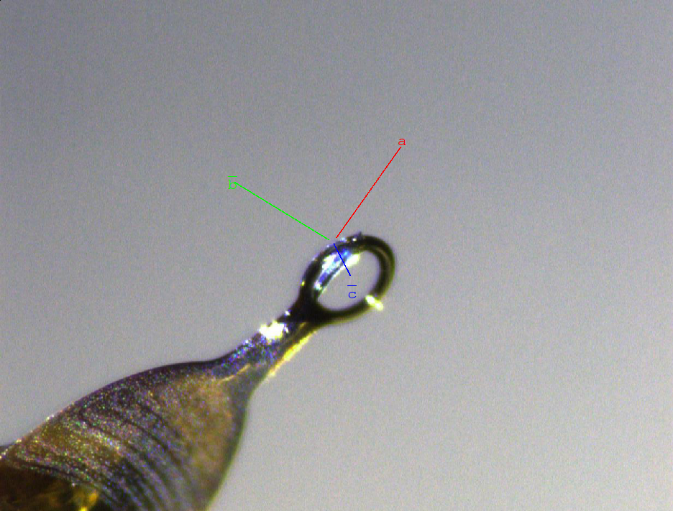 |

**Figure S6 a** Crystal views of complex **3**


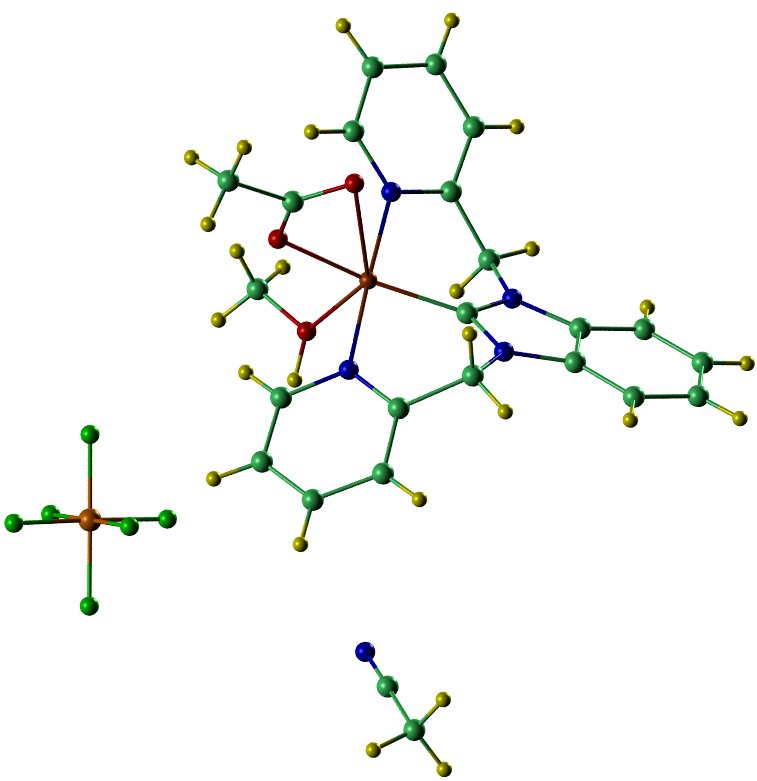


**Figure S6 b** Figure showing the asymmetric unit of complex **3**

| ***Table S6 a. Sample and crystal data for complex 3.***  ____________________________________________________________________________________________ |
| --- |

| **Identification code** | C_JE_002_100K | |
| --- | --- | --- |
| **Chemical formula** | C_24_H_26_CuF_6_N_5_O_3_P | |
| **Formula weight** | 641.01 g/mol | |
| **Temperature** | 100(2) K | |
| **Wavelength** | 1.54178 Å | |
| **Crystal size** | (0.080 x 0.142 x 0.182) mm^3^ | |
| **Crystal system** | monoclinic | |
| **Space group** | P 1 2(1)/n 1 | |
| **Unit cell dimensions** | a = 6.9851(3) Å | α = 90° |
|  | b = 26.4631(10) Å | β = 94.430(3)° |
|  | c = 14.4471(6) Å | γ = 90° |
| **Volume** | 2662.53(19) Å^3^ |  |
| **Z** | 4 | |
| **Density (calculated)** | 1.599 g/cm^3^ | |
| **Absorption coefficient** | 2.447 mm^-1^ | |
| **F(000)** | 1308 | |

| ***Table S6 b. Data collection and structure refinement for complex 3.***  ____________________________________________________________________________________________ |
| --- |

| **Theta range for data collection** | 3.34 to 68.25° | |
| --- | --- | --- |
| **Index ranges** | -7<=h<=8, -31<=k<=31, -17<=l<=17 | |
| **Reflections collected** | 30831 | |
| **Independent reflections** | 4842 [R(int) = 0.2554] | |
| **Coverage of independent reflections** | 99.1% | |
| **Absorption correction** | Multi-Scan | |
| **Max. and min. transmission** | 0.8280 and 0.6640 | |
| **Refinement method** | Full-matrix least-squares on F^2^ | |
| **Refinement program** | SHELXL-2018/3 (Sheldrick, 2018) | |
| **Function minimized** | Σ w(F_o_^2^ - F_c_^2^)^2^ | |
| **Data / restraints / parameters** | 4842 / 285 / 429 | |
| **Goodness-of-fit on F^2^** | 1.132 | |
| **Final R indices** | 3138 data; I>2σ(I) | R1 = 0.1072, wR2 = 0.1900 |
|  | all data | R1 = 0.1712, wR2 = 0.2198 |
| **Weighting scheme** | w=1/[σ^2^(F_o_^2^)+32.4747P] where P=(F_o_^2^+2F_c_^2^)/3 | |
| **Extinction coefficient** | 0.0002(1) | |
| **Largest diff. peak and hole** | 0.590 and -0.669 eÅ^-3^ | |
| **R.M.S. deviation from mean** | 0.131 eÅ^-3^ | |

| ***Table S6 c. Atomic coordinates and equivalent isotropic atomic displacement parameters (Å^2^) for complex 3.***  ____________________________________________________________________________________________ |
| --- |
| ***U(eq) is defined as one third of the trace of the orthogonalized U_ij_ tensor.*** |
|  |

|  | **x/a** | **y/b** | **z/c** | **U(eq)** |
| --- | --- | --- | --- | --- |
| Cu1 | 0.24771(17) | 0.35664(4) | 0.15227(8) | 0.0187(3) |
| N1 | 0.2734(9) | 0.4217(2) | 0.9893(4) | 0.0151(14) |
| N2 | 0.2384(9) | 0.4655(2) | 0.1132(4) | 0.0177(14) |
| N3 | 0.3559(10) | 0.3972(3) | 0.2667(4) | 0.0206(15) |
| N4 | 0.1467(10) | 0.3170(3) | 0.0314(5) | 0.0201(15) |
| C1 | 0.2551(11) | 0.4186(3) | 0.0815(5) | 0.0184(17) |
| C2 | 0.2657(11) | 0.4722(3) | 0.9612(5) | 0.0174(17) |
| C3 | 0.2778(11) | 0.4955(3) | 0.8754(6) | 0.0218(18) |
| C4 | 0.2599(12) | 0.5470(3) | 0.8720(6) | 0.0242(19) |
| C5 | 0.2329(12) | 0.5757(3) | 0.9518(6) | 0.026(2) |
| C6 | 0.2246(12) | 0.5528(3) | 0.0383(6) | 0.0231(19) |
| C7 | 0.2411(12) | 0.5005(3) | 0.0411(6) | 0.0216(18) |
| C8 | 0.2108(12) | 0.4766(3) | 0.2100(5) | 0.0231(19) |
| C9 | 0.3469(11) | 0.4475(3) | 0.2766(5) | 0.0169(17) |
| C10 | 0.4452(12) | 0.4730(3) | 0.3501(6) | 0.0241(19) |
| C11 | 0.5584(14) | 0.4449(4) | 0.4147(6) | 0.029(2) |
| C12 | 0.5698(13) | 0.3939(4) | 0.4045(6) | 0.028(2) |
| C13 | 0.4711(13) | 0.3708(3) | 0.3292(5) | 0.0237(19) |
| C14 | 0.3035(12) | 0.3776(3) | 0.9319(5) | 0.0200(17) |
| C15 | 0.1654(12) | 0.3363(3) | 0.9446(6) | 0.0203(18) |
| C16 | 0.0594(12) | 0.3157(3) | 0.8684(6) | 0.0245(19) |
| C17 | 0.9430(13) | 0.2747(4) | 0.8772(6) | 0.029(2) |
| C18 | 0.9354(13) | 0.2538(3) | 0.9655(6) | 0.030(2) |
| C19 | 0.0348(12) | 0.2766(3) | 0.0403(6) | 0.0221(18) |
| P1A | 0.6002(9) | 0.3658(2) | 0.7016(4) | 0.0331(11) |
| F1A | 0.5982(17) | 0.3956(4) | 0.6050(6) | 0.041(2) |
| F2A | 0.4730(17) | 0.4096(4) | 0.7409(7) | 0.0400(19) |
| F3A | 0.4116(14) | 0.3356(4) | 0.6674(9) | 0.0488(19) |
| F4A | 0.7298(15) | 0.3224(4) | 0.6624(8) | 0.045(2) |
| F5A | 0.7900(15) | 0.3961(4) | 0.7372(8) | 0.0395(18) |
| F6A | 0.6053(18) | 0.3378(4) | 0.7989(7) | 0.0414(19) |
| P1B | 0.5707(8) | 0.3633(2) | 0.6900(4) | 0.0332(11) |
| F1B | 0.6420(16) | 0.4053(4) | 0.6206(6) | 0.0377(19) |
| F2B | 0.3930(15) | 0.3983(4) | 0.7093(8) | 0.0381(19) |
| F3B | 0.4448(15) | 0.3365(4) | 0.6079(7) | 0.0391(18) |
| F4B | 0.7495(14) | 0.3284(4) | 0.6713(8) | 0.041(2) |
| F5B | 0.6953(19) | 0.3903(4) | 0.7732(7) | 0.0432(18) |
| F6B | 0.5003(18) | 0.3227(4) | 0.7607(8) | 0.0469(19) |
| O1S | 0.1709(8) | 0.3043(2) | 0.2395(4) | 0.0195(12) |
| O2S | 0.8907(9) | 0.3434(2) | 0.2176(4) | 0.0256(14) |
| C2S | 0.9997(12) | 0.3132(3) | 0.2596(6) | 0.0201(18) |
| C3S | 0.9347(13) | 0.2843(4) | 0.3431(6) | 0.033(2) |
| O3S | 0.5378(9) | 0.3179(2) | 0.1417(4) | 0.0271(14) |
| C1S | 0.5458(14) | 0.2652(3) | 0.1245(7) | 0.031(2) |
| N2S | 0.9547(15) | 0.5078(4) | 0.3736(7) | 0.055(3) |
| C4S | 0.9448(15) | 0.5447(5) | 0.4119(8) | 0.043(3) |
| C5S | 0.9276(16) | 0.5913(5) | 0.4646(8) | 0.052(3) |

| ***Table S6 d. Bond lengths (Å) for complex 3.***  ____________________________________________________________________________________________ |
| --- |

| Cu1-C1 | 1.935(8) | Cu1-O1S | 1.975(5) |  |
| --- | --- | --- | --- | --- |
| Cu1-N3 | 2.066(7) | Cu1-N4 | 2.112(7) |  |
| Cu1-O3S | 2.287(6) | N1-C1 | 1.350(10) |  |
| N1-C2 | 1.395(10) | N1-C14 | 1.458(10) |  |
| N2-C1 | 1.332(10) | N2-C7 | 1.395(10) |  |
| N2-C8 | 1.456(10) | N3-C9 | 1.340(10) |  |
| N3-C13 | 1.357(10) | N4-C19 | 1.337(11) |  |
| N4-C15 | 1.370(10) | C2-C3 | 1.393(11) |  |
| C2-C7 | 1.397(11) | C3-C4 | 1.369(12) |  |
| C4-C5 | 1.407(12) | C5-C6 | 1.393(12) |  |
| C6-C7 | 1.390(12) | C8-C9 | 1.510(11) |  |
| C9-C10 | 1.394(11) | C10-C11 | 1.392(12) |  |
| C11-C12 | 1.361(13) | C12-C13 | 1.384(11) |  |
| C14-C15 | 1.478(11) | C15-C16 | 1.390(11) |  |
| C16-C17 | 1.368(13) | C17-C18 | 1.396(12) |  |
| C18-C19 | 1.376(12) | P1A-F6A | 1.587(8) |  |
| P1A-F3A | 1.587(9) | P1A-F2A | 1.590(8) |  |
| P1A-F4A | 1.595(7) | P1A-F5A | 1.599(9) |  |
| P1A-F1A | 1.602(7) | P1B-F6B | 1.586(8) |  |
| P1B-F3B | 1.589(9) | P1B-F2B | 1.591(8) |  |
| P1B-F4B | 1.594(7) | P1B-F5B | 1.597(9) |  |
| P1B-F1B | 1.603(7) | O1S-C2S | 1.274(10) |  |
| O2S-C2S | 1.231(10) | C2S-C3S | 1.527(12) |  |
| O3S-C1S | 1.418(10) | N2S-C4S | 1.128(14) |  |
| C4S-C5S | 1.458(17) |  |  |  |
| ***Table S6 e. Bond angles (°) for complex 3.***  *____________________________________________________________________________________________* | | | | |

| C1-Cu1-O1S | 162.1(3) | C1-Cu1-N3 | 87.7(3) |
| --- | --- | --- | --- |
| O1S-Cu1-N3 | 87.4(2) | C1-Cu1-N4 | 90.3(3) |
| O1S-Cu1-N4 | 95.1(2) | N3-Cu1-N4 | 177.2(3) |
| C1-Cu1-O3S | 106.6(3) | O1S-Cu1-O3S | 90.8(2) |
| N3-Cu1-O3S | 90.9(2) | N4-Cu1-O3S | 87.9(2) |
| C1-N1-C2 | 110.0(6) | C1-N1-C14 | 122.7(6) |
| C2-N1-C14 | 127.3(6) | C1-N2-C7 | 110.8(7) |
| C1-N2-C8 | 122.6(7) | C7-N2-C8 | 126.5(7) |
| C9-N3-C13 | 118.2(7) | C9-N3-Cu1 | 125.7(5) |
| C13-N3-Cu1 | 115.2(5) | C19-N4-C15 | 119.0(7) |
| C19-N4-Cu1 | 118.6(6) | C15-N4-Cu1 | 121.5(5) |
| N2-C1-N1 | 107.4(7) | N2-C1-Cu1 | 127.0(6) |
| N1-C1-Cu1 | 125.6(6) | C3-C2-N1 | 132.8(7) |
| C3-C2-C7 | 121.2(8) | N1-C2-C7 | 106.1(7) |
| C4-C3-C2 | 117.4(8) | C3-C4-C5 | 121.8(8) |
| C6-C5-C4 | 121.2(8) | C7-C6-C5 | 116.7(8) |
| C6-C7-N2 | 132.6(8) | C6-C7-C2 | 121.7(8) |
| N2-C7-C2 | 105.7(7) | N2-C8-C9 | 112.7(7) |
| N3-C9-C10 | 122.5(7) | N3-C9-C8 | 118.1(7) |
| C10-C9-C8 | 119.1(7) | C11-C10-C9 | 118.2(8) |
| C12-C11-C10 | 119.5(8) | C11-C12-C13 | 119.6(8) |
| N3-C13-C12 | 121.9(8) | N1-C14-C15 | 113.4(6) |
| N4-C15-C16 | 119.7(8) | N4-C15-C14 | 119.9(7) |
| C16-C15-C14 | 120.3(8) | C17-C16-C15 | 121.4(8) |
| C16-C17-C18 | 117.6(8) | C19-C18-C17 | 119.5(8) |
| N4-C19-C18 | 122.5(8) | F6A-P1A-F3A | 90.1(6) |
| F6A-P1A-F2A | 89.9(5) | F3A-P1A-F2A | 90.3(6) |
| F6A-P1A-F4A | 90.2(5) | F3A-P1A-F4A | 90.4(5) |
| F2A-P1A-F4A | 179.4(7) | F6A-P1A-F5A | 89.3(6) |
| F3A-P1A-F5A | 179.4(7) | F2A-P1A-F5A | 89.6(6) |
| F4A-P1A-F5A | 89.8(5) | F6A-P1A-F1A | 178.3(6) |
| F3A-P1A-F1A | 91.4(6) | F2A-P1A-F1A | 89.2(4) |
| F4A-P1A-F1A | 90.7(4) | F5A-P1A-F1A | 89.2(5) |
| F6B-P1B-F3B | 89.8(6) | F6B-P1B-F2B | 89.9(5) |
| F3B-P1B-F2B | 89.8(6) | F6B-P1B-F4B | 90.1(5) |
| F3B-P1B-F4B | 90.5(5) | F2B-P1B-F4B | 179.6(7) |
| F6B-P1B-F5B | 89.9(6) | F3B-P1B-F5B | 179.4(7) |
| F2B-P1B-F5B | 89.7(6) | F4B-P1B-F5B | 90.0(5) |
| F6B-P1B-F1B | 178.7(6) | F3B-P1B-F1B | 91.3(6) |
| F2B-P1B-F1B | 89.4(4) | F4B-P1B-F1B | 90.6(4) |
| F5B-P1B-F1B | 89.0(6) | C2S-O1S-Cu1 | 108.7(5) |
| O2S-C2S-O1S | 124.3(8) | O2S-C2S-C3S | 120.3(8) |
| O1S-C2S-C3S | 115.4(7) | C1S-O3S-Cu1 | 120.0(5) |
| N2S-C4S-C5S | 177.5(12) |  |  |

| ***Table S6 f. Torsion angles (°) for complex 3.***  *____________________________________________________________________________________________* |
| --- |

| C7-N2-C1-N1 | 1.3(9) | C8-N2-C1-N1 | 178.3(7) |
| --- | --- | --- | --- |
| C7-N2-C1-Cu1 | -177.0(6) | C8-N2-C1-Cu1 | -0.1(11) |
| C2-N1-C1-N2 | -0.7(9) | C14-N1-C1-N2 | 177.2(7) |
| C2-N1-C1-Cu1 | 177.7(6) | C14-N1-C1-Cu1 | -4.4(10) |
| C1-N1-C2-C3 | 179.8(8) | C14-N1-C2-C3 | 1.9(14) |
| C1-N1-C2-C7 | -0.1(9) | C14-N1-C2-C7 | -178.0(7) |
| N1-C2-C3-C4 | 178.5(8) | C7-C2-C3-C4 | -1.6(12) |
| C2-C3-C4-C5 | 0.8(13) | C3-C4-C5-C6 | 0.5(13) |
| C4-C5-C6-C7 | -1.0(12) | C5-C6-C7-N2 | -179.6(8) |
| C5-C6-C7-C2 | 0.3(12) | C1-N2-C7-C6 | 178.5(9) |
| C8-N2-C7-C6 | 1.7(14) | C1-N2-C7-C2 | -1.4(9) |
| C8-N2-C7-C2 | -178.2(7) | C3-C2-C7-C6 | 1.1(12) |
| N1-C2-C7-C6 | -179.0(7) | C3-C2-C7-N2 | -179.0(7) |
| N1-C2-C7-N2 | 0.9(8) | C1-N2-C8-C9 | 46.8(10) |
| C7-N2-C8-C9 | -136.8(8) | C13-N3-C9-C10 | -2.5(12) |
| Cu1-N3-C9-C10 | -171.1(6) | C13-N3-C9-C8 | -176.8(7) |
| Cu1-N3-C9-C8 | 14.5(10) | N2-C8-C9-N3 | -52.7(10) |
| N2-C8-C9-C10 | 132.8(8) | N3-C9-C10-C11 | 0.8(13) |
| C8-C9-C10-C11 | 175.1(8) | C9-C10-C11-C12 | 0.0(13) |
| C10-C11-C12-C13 | 1.0(14) | C9-N3-C13-C12 | 3.5(13) |
| Cu1-N3-C13-C12 | 173.3(7) | C11-C12-C13-N3 | -2.8(14) |
| C1-N1-C14-C15 | 50.1(10) | C2-N1-C14-C15 | -132.3(8) |
| C19-N4-C15-C16 | 3.9(11) | Cu1-N4-C15-C16 | -164.8(6) |
| C19-N4-C15-C14 | -173.6(7) | Cu1-N4-C15-C14 | 17.6(10) |
| N1-C14-C15-N4 | -55.8(10) | N1-C14-C15-C16 | 126.7(8) |
| N4-C15-C16-C17 | -3.2(13) | C14-C15-C16-C17 | 174.3(8) |
| C15-C16-C17-C18 | -0.6(14) | C16-C17-C18-C19 | 3.7(14) |
| C15-N4-C19-C18 | -0.8(12) | Cu1-N4-C19-C18 | 168.3(7) |
| C17-C18-C19-N4 | -3.1(14) | Cu1-O1S-C2S-O2S | 12.7(10) |
| Cu1-O1S-C2S-C3S | -165.9(6) |  |  |

| ***Table S6 g. Anisotropic atomic displacement parameters (Å^2^) for complex 3.***  ____________________________________________________________________________________________ |
| --- |
| ***The anisotropic atomic displacement factor exponent takes the form: -2π^2^[ h^2^ a^*2^ U_11_ + ... + 2 h k a^*^ b^*^ U_12_ ]*** |

|  | **U_11_** | **U_22_** | **U_33_** | **U_23_** | **U_13_** | **U_12_** |
| --- | --- | --- | --- | --- | --- | --- |
| Cu1 | 0.0260(6) | 0.0163(6) | 0.0130(6) | 0.0017(5) | -0.0039(4) | -0.0009(5) |
| N1 | 0.014(3) | 0.015(3) | 0.016(3) | 0.003(3) | 0.001(3) | -0.002(3) |
| N2 | 0.020(4) | 0.017(3) | 0.015(3) | 0.001(3) | -0.004(3) | -0.003(3) |
| N3 | 0.024(4) | 0.024(4) | 0.013(3) | 0.001(3) | -0.003(3) | -0.003(3) |
| N4 | 0.022(4) | 0.020(4) | 0.018(3) | -0.001(3) | -0.003(3) | -0.002(3) |
| C1 | 0.022(4) | 0.018(4) | 0.015(4) | 0.005(3) | -0.004(3) | -0.005(3) |
| C2 | 0.017(4) | 0.018(4) | 0.016(4) | 0.002(3) | -0.001(3) | -0.002(3) |
| C3 | 0.017(4) | 0.027(5) | 0.021(4) | -0.002(4) | -0.002(3) | 0.001(3) |
| C4 | 0.029(5) | 0.024(5) | 0.020(4) | 0.007(3) | 0.002(4) | 0.005(4) |
| C5 | 0.014(4) | 0.025(5) | 0.037(5) | 0.010(4) | -0.001(4) | -0.006(3) |
| C6 | 0.021(4) | 0.019(4) | 0.028(5) | 0.000(4) | -0.010(4) | 0.003(3) |
| C7 | 0.021(4) | 0.020(4) | 0.023(4) | 0.004(4) | -0.003(3) | -0.003(3) |
| C8 | 0.030(5) | 0.024(5) | 0.014(4) | -0.001(4) | -0.006(3) | 0.002(4) |
| C9 | 0.021(4) | 0.021(4) | 0.008(4) | 0.006(3) | 0.001(3) | 0.001(3) |
| C10 | 0.023(5) | 0.030(5) | 0.018(4) | -0.005(4) | -0.006(3) | 0.000(4) |
| C11 | 0.039(5) | 0.033(5) | 0.015(4) | 0.004(4) | 0.000(4) | -0.003(4) |
| C12 | 0.037(5) | 0.035(5) | 0.009(4) | 0.001(4) | -0.009(4) | 0.004(4) |
| C13 | 0.042(5) | 0.021(5) | 0.007(4) | 0.003(3) | -0.006(3) | 0.000(4) |
| C14 | 0.023(4) | 0.021(4) | 0.016(4) | 0.002(3) | 0.001(3) | -0.002(3) |
| C15 | 0.023(4) | 0.015(4) | 0.022(4) | -0.010(3) | -0.001(3) | 0.005(3) |
| C16 | 0.032(5) | 0.027(5) | 0.014(4) | -0.004(4) | -0.003(3) | 0.001(4) |
| C17 | 0.030(5) | 0.041(6) | 0.015(4) | -0.007(4) | -0.002(4) | -0.007(4) |
| C18 | 0.034(5) | 0.025(5) | 0.031(5) | -0.007(4) | 0.003(4) | -0.011(4) |
| C19 | 0.024(4) | 0.019(4) | 0.023(4) | -0.002(3) | -0.002(3) | 0.001(3) |
| P1A | 0.052(2) | 0.0235(19) | 0.024(2) | 0.0009(17) | 0.0049(18) | 0.0043(18) |
| F1A | 0.063(5) | 0.038(4) | 0.022(3) | 0.000(3) | 0.004(3) | -0.002(4) |
| F2A | 0.060(4) | 0.030(4) | 0.031(4) | 0.006(3) | 0.012(4) | 0.010(3) |
| F3A | 0.057(3) | 0.040(4) | 0.047(4) | 0.003(4) | -0.003(3) | -0.006(3) |
| F4A | 0.061(4) | 0.034(4) | 0.039(4) | -0.010(3) | 0.004(4) | 0.009(4) |
| F5A | 0.054(4) | 0.035(4) | 0.029(4) | -0.001(3) | 0.002(3) | -0.005(3) |
| F6A | 0.062(5) | 0.032(4) | 0.031(3) | 0.009(3) | 0.006(3) | 0.006(3) |
| P1B | 0.052(2) | 0.025(2) | 0.023(2) | -0.0021(17) | 0.0046(18) | 0.0054(18) |
| F1B | 0.059(4) | 0.032(4) | 0.022(3) | -0.004(3) | 0.005(3) | -0.004(3) |
| F2B | 0.056(4) | 0.031(4) | 0.027(4) | -0.006(3) | 0.006(3) | 0.008(3) |
| F3B | 0.053(4) | 0.033(4) | 0.030(3) | -0.012(3) | 0.002(3) | -0.001(3) |
| F4B | 0.056(4) | 0.034(4) | 0.034(4) | -0.010(3) | 0.001(3) | 0.009(3) |
| F5B | 0.065(4) | 0.038(4) | 0.026(3) | -0.008(3) | -0.002(3) | 0.004(3) |
| F6B | 0.069(5) | 0.034(4) | 0.039(4) | 0.008(3) | 0.010(4) | 0.002(3) |
| O1S | 0.023(3) | 0.018(3) | 0.016(3) | 0.006(2) | -0.005(2) | -0.001(2) |
| O2S | 0.034(3) | 0.024(3) | 0.018(3) | 0.004(2) | -0.003(2) | 0.003(3) |
| C2S | 0.022(4) | 0.018(4) | 0.020(4) | -0.002(3) | -0.005(3) | -0.004(3) |
| C3S | 0.027(5) | 0.045(6) | 0.028(5) | 0.015(4) | 0.005(4) | 0.002(4) |
| O3S | 0.029(3) | 0.020(3) | 0.030(3) | -0.002(3) | -0.004(3) | 0.003(3) |
| C1S | 0.035(5) | 0.026(5) | 0.033(5) | -0.008(4) | 0.006(4) | 0.002(4) |
| N2S | 0.053(6) | 0.071(7) | 0.042(6) | -0.016(5) | 0.013(5) | -0.008(5) |
| C4S | 0.032(6) | 0.060(8) | 0.037(6) | 0.002(6) | 0.005(5) | -0.009(5) |
| C5S | 0.037(6) | 0.071(9) | 0.050(7) | 0.002(6) | 0.009(5) | -0.003(6) |

| ***Table S6 h. Hydrogen atomic coordinates and isotropic atomic displacement parameters (Å^2^) for complex 3.***  ____________________________________________________________________________________________ |
| --- |

|  | **x/a** | **y/b** | **z/c** | **U(eq)** |
| --- | --- | --- | --- | --- |
| H3 | 0.2978 | 0.4764 | -0.1787 | 0.026000 |
| H4 | 0.2659 | 0.5637 | -0.1859 | 0.029000 |
| H5 | 0.2201 | 0.6114 | -0.0533 | 0.031000 |
| H6 | 0.2085 | 0.5721 | 0.0927 | 0.028000 |
| H8A | 0.0770 | 0.4682 | 0.2225 | 0.028000 |
| H8B | 0.2297 | 0.5132 | 0.2211 | 0.028000 |
| H10 | 0.4353 | 0.5087 | 0.3559 | 0.029000 |
| H11 | 0.6271 | 0.4612 | 0.4656 | 0.035000 |
| H12 | 0.6451 | 0.3743 | 0.4487 | 0.033000 |
| H13 | 0.4844 | 0.3354 | 0.3211 | 0.028000 |
| H14A | 0.4353 | 0.3647 | -0.0531 | 0.024000 |
| H14B | 0.2930 | 0.3879 | -0.1342 | 0.024000 |
| H16 | 0.0680 | 0.3304 | -0.1911 | 0.029000 |
| H17 | -0.1301 | 0.2609 | -0.1750 | 0.034000 |
| H18 | -0.1378 | 0.2242 | -0.0260 | 0.036000 |
| H19 | 0.0235 | 0.2630 | 0.1005 | 0.027000 |
| H3S1 | 0.0078 | 0.2960 | 0.3997 | 0.050000 |
| H3S2 | -0.0431 | 0.2481 | 0.3348 | 0.050000 |
| H3S3 | -0.2025 | 0.2903 | 0.3484 | 0.050000 |
| H1S1 | 0.6657 | 0.2514 | 0.1540 | 0.047000 |
| H1S2 | 0.5410 | 0.2592 | 0.0574 | 0.047000 |
| H1S3 | 0.4364 | 0.2485 | 0.1502 | 0.047000 |
| H5S1 | 0.7954 | 0.6038 | 0.4559 | 0.078000 |
| H5S2 | 1.0154 | 0.6167 | 0.4426 | 0.078000 |
| H5S3 | 0.9605 | 0.5846 | 0.5306 | 0.078000 |
| H1S | 0.6271 | 0.3338 | 0.1869 | 0.078000 |

**Table S6 i.** Selected bond lengths (Å) bond angles (°) for complex **3**

| Bond Length (Å) | Complex 3 |
| --- | --- |
| Cu1-C1 | 1.935(8) |
| Cu1-N4 | 2.112(7) |
| Cu1-N3 | 2.066(7) |
| Cu1-O3S | 2.287(6) |
| Cu1-O1S | 1.975(5) |
| Cu1-O2S | 2.758 |
| Bond Angles (°) | |
| N3-Cu1-N4 | 177.2(3) |
| O1S-Cu1-C1 | 162.1(3) |
| N3-Cu1-C1 | 87.7(3) |
| N3-Cu1-O3S | 90.9(2) |
| N3-Cu1-O2S | 94.40 |

The best crystal we were able to select from the batch was a very small plate: (0.18*0.14*0.08) mm^3^ (Figure S6 c). This crystal shows quite poor and diffuse diffraction at high Bragg angles (Figure S6 d). The data was measured and integrated in a monoclinic unit cell; Centrosymmetric Space group: P 2(1)/n. The range for data collection was 6.68 - 149.0°; 30831 reflections were collected with a coverage of independent reflections of 99.1%; average redundancy 6.367. The integration of the data yielded a total of 30831 reflections to a maximum θ angle of 68.25° (0.83 Å resolution), of which 4842 were independent (R_int_ = 25.54%, R_sig_ = 11.74%) and 3138 (64.81%) were greater than 2σ (F^2^). The overall quality of the structure is acceptable for publication according to the fact the refinement does not show any issues owing the checkcif: (Two alert A concerning the value of Rint greater than 0.25 and the value of Rint is greater than 0.12). The goodness-of-fit: GOF was 1.132 that is an acceptable value for this parameter. The largest peak in the final difference electron density synthesis was 0.590 e^-^/Å^3^ and the largest hole was -0.669 e^-^/Å^3^ with an RMS deviation of 0.131 e^-^/Å^3^. Meaning all significant electronic density peaks were correctly assigned and refined. All heavy atoms have been correctly anisotropically refined. The model was refined without any constraints put on ADP’s parameters or restraints applied on angles and interatomic lengths excepted for the disordered part. A PF_6_^-^ anion that was found statistically distributed on two positions and anisotropically refined with a ratio of occupancy equal to: 50%. A molecule of acetate and methanol were also localized coordinated on the metal center and a free molecule of acetonitrile as well. A certainly better data set could be obtained using a more powerful X-ray source such as, a Copper X-ray radiation using a rotating anode or even a synchrotron radiation. In all case, the crystal structure determination reported in this manuscript was the best, which was possible to obtain owing the relatively poor diffracted intensity measured using standard single-crystal techniques. All attempts to try to grow bigger crystals using different methods and solvents failed.

| 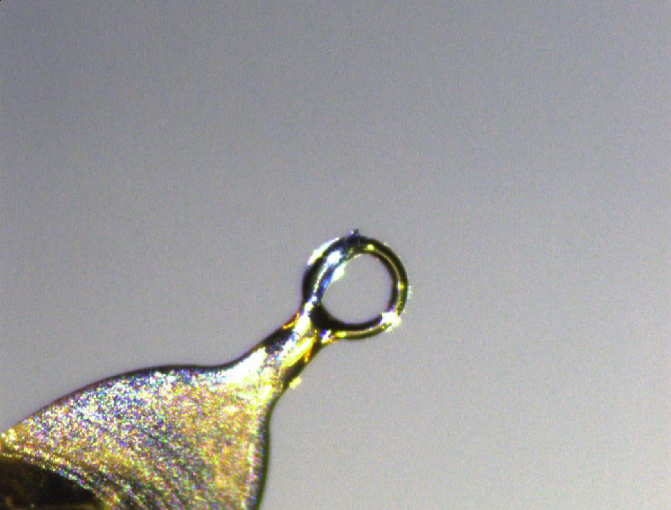 | 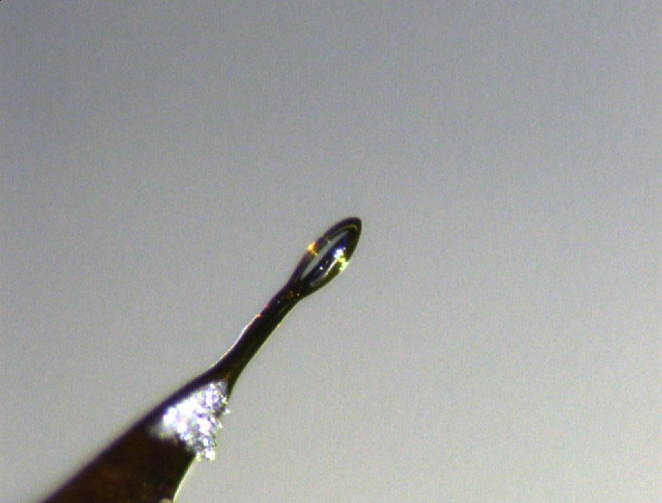 |
| --- | --- |
| 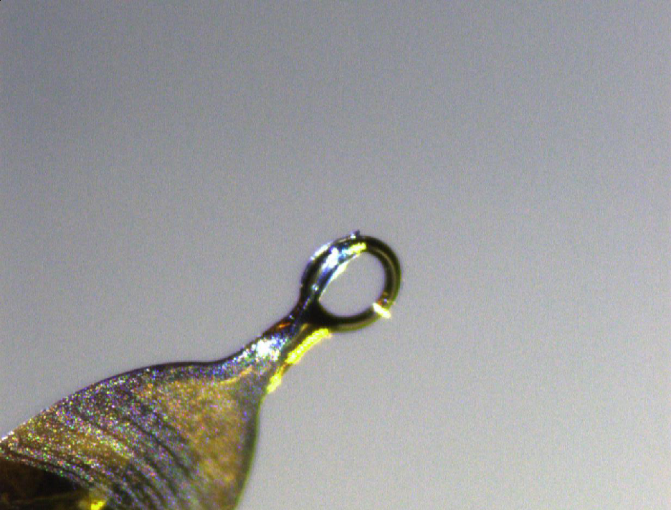 | 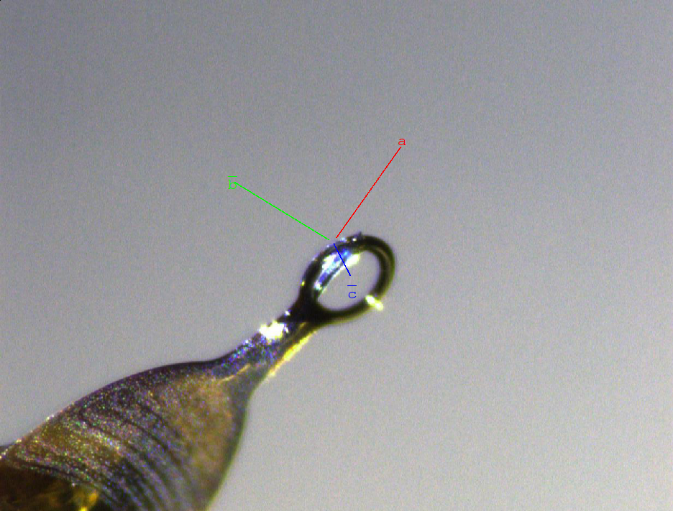 |

**Figure S6 c** Pictures of the measured crystal: platelet with the following dimensions:

(0.080x 0.142 x 0.182) mm^3^


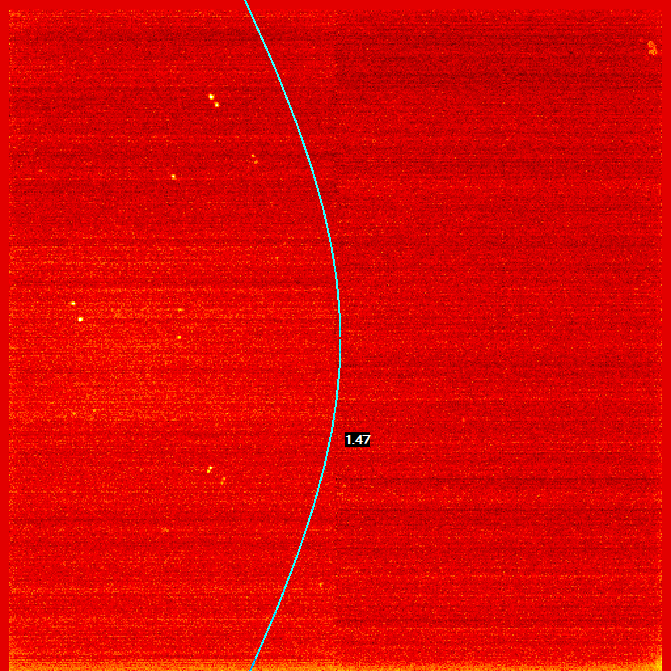


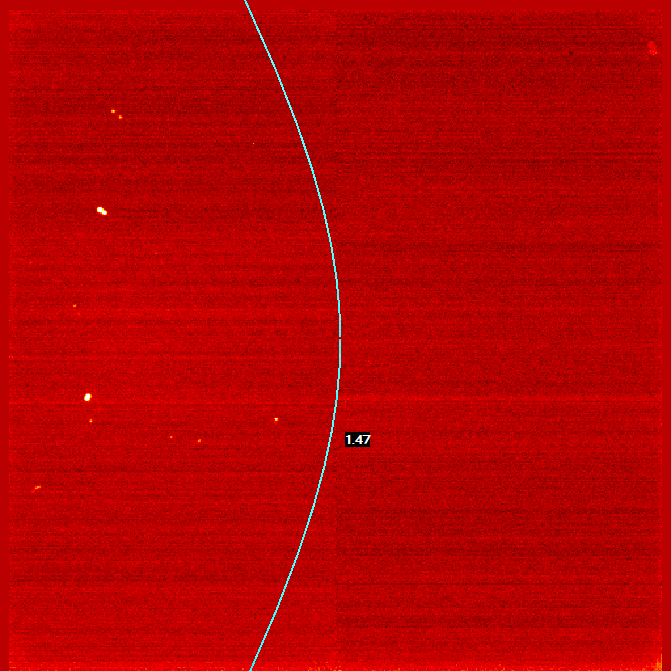


**Figure S6 d** Diffraction Pattern Frames: Showing no significant diffracted intensity above 1.3 Å of resolution, using a monochromatized Cu X-ray radiation

**Powder X-ray diffraction study**

Powder X-ray diffraction patterns on complex **3** and compound **2** only were performed at room temperature, between 5° and 50° in 2theta using an AXRD Benchtop X-Ray system from Proto Manufacturing equipped with a powerful hybrid photon-counting detector: DECTRIS MYTHEN2.R.1D and using a monochromatized Copper X-ray radiation (λ = 1.54178 Å). A simulated powder X-ray diffraction pattern was also created using the unit cell and data measured from the X-ray single crystal analysis of **3** excluding all solvents and hydrogen atoms of the model analyzed (https://www.ccdc.cam.ac.uk/). We observed that in the region between 5° and 30° in 2theta Bragg angles, that the data are comparable. Indeed, it makes reasonable to write the analyzed powder contains the same complex **3** like the one localized and refined by single X-ray diffraction methods (Table S7). Discrepancies observed between two diffraction powder patterns can be attributed to the solvents used during the process of crystallization.

**Table S7** Table showing 2theta values for complex **3** powder XRD (measured and simulated)

| 2theta for complex 3 powder  XRD pattern (Measured) | 2theta for complex 3 powder  XRD pattern (Simulated)* | |
| --- | --- | --- |
| 6.96° | 7.23° |  |
| 7.23° | 8.28° |  |
| 12.40° | 12.6° |  |
| 15.71° | 15.78° |  |
| 19.82° | 20.09° |  |
| 21.0° | 21.1° |  |
| 26.05° | 26.08° |  |

*Excluding solvents of crystallization


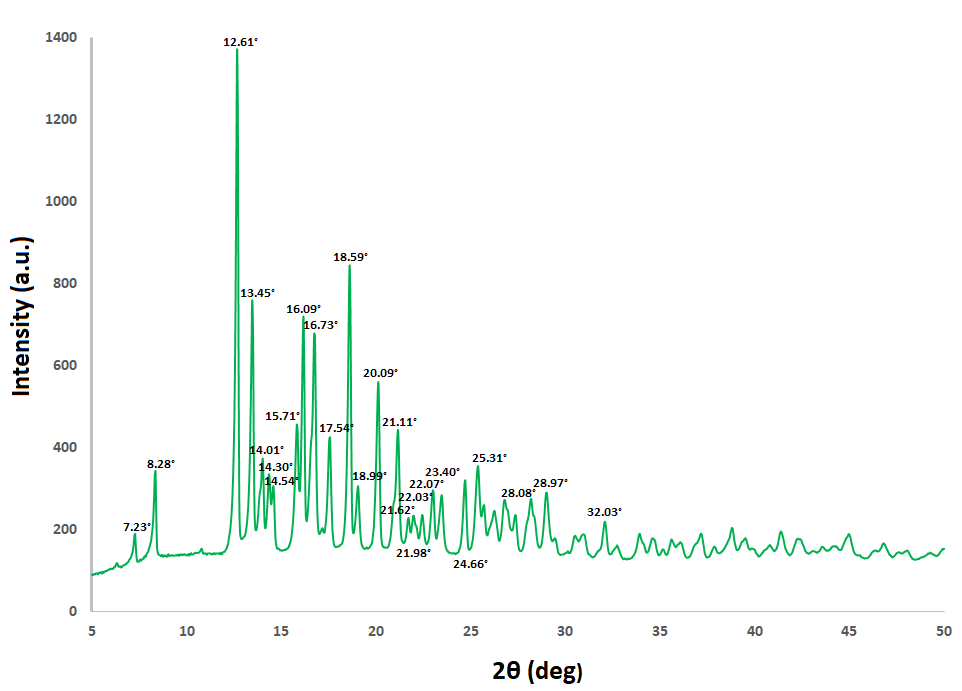


**Figure S7 a** Complex **3** powder XRD pattern (measured).


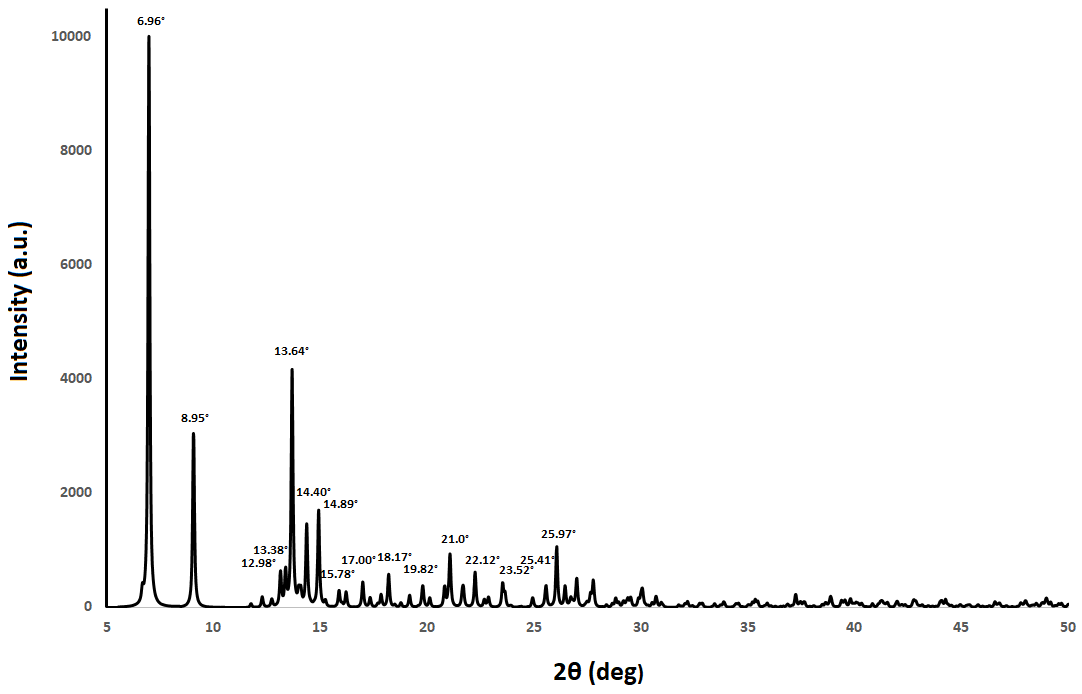


**Figure S7 b** Complex **3** powder XRD pattern (simulated).


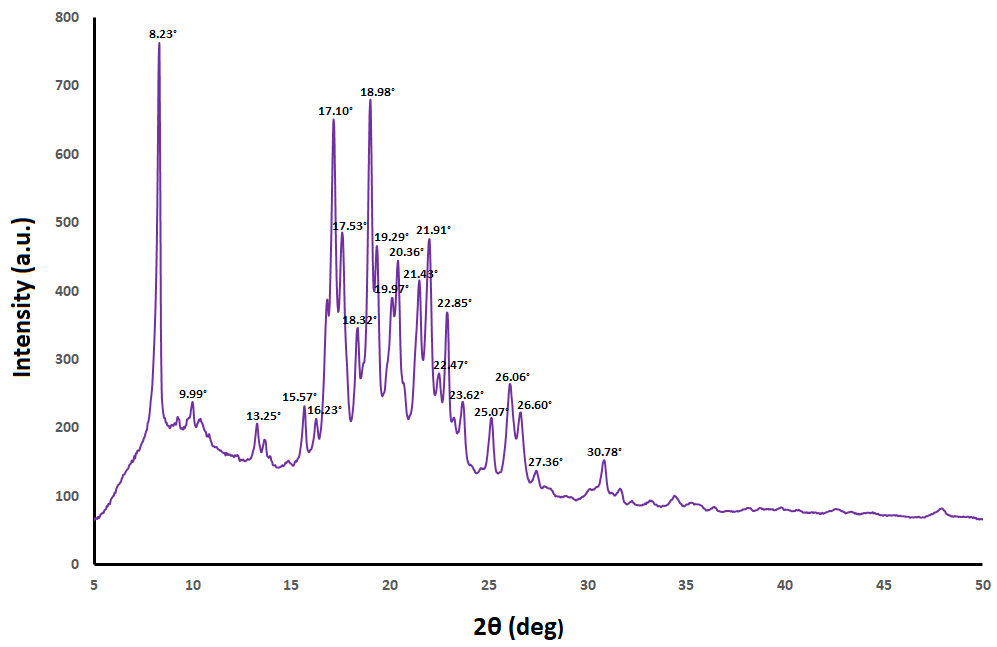


**Figure S7 c** Compound **2** powder XRD pattern (measured).


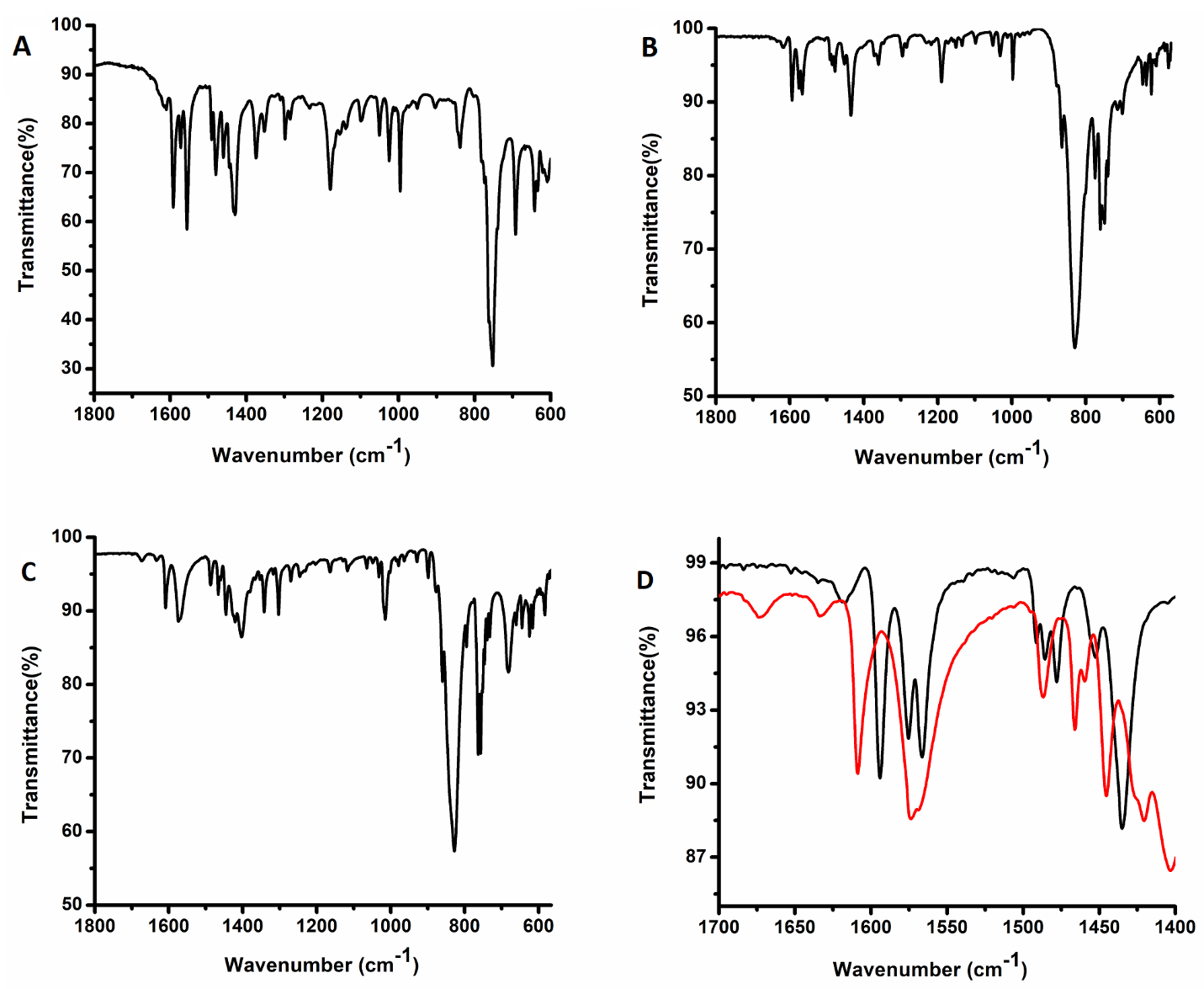


**Figure S8** FT-IR spectra of (A) compound **1** (B) compound **2** (c) complex **3** (D) comparison of compound **2** (black) and complex **3** (red).
